# Cyclic di-GMP suppresses cancer metastasis by targeting proteasome 26S subunit non-ATPase 3 independently of STING

**DOI:** 10.1038/s41392-025-02553-9

**Published:** 2026-02-04

**Authors:** Jieqiong Wang, Alexander Mrozek, Kewen Hu, Hanyu You, Sarah E. Traverse, Hyemin Lee, Shelya X. Zeng, Xiufeng Pang, Heewon Park, Hua Lu

**Affiliations:** 1 Department of Biochemistry & Molecular Biology, Tulane University School of Medicine, New Orleans, LA USA; 2 Tulane Cancer Center, Tulane University School of Medicine, New Orleans, LA USA; 3 Shanghai Key Laboratory of Regulatory Biology, School of Life Sciences, East China Normal University, Shanghai, China; 4 Department of Radiation Oncology, Fudan University Shanghai Cancer Center, Shanghai, China

**Keywords:** Breast cancer, Target identification

## Abstract

Cancer metastasis is the primary cause of cancer-related mortality, yet effective treatments remain limited. There is an urgent need to develop novel therapeutic strategies to combat metastasis. In this study, we demonstrate that the bacterial intracellular signaling molecule cyclic di-GMP (c-di-GMP, or cdG) exerts a potent inhibitory effect on cancer metastasis, particularly in metastatic breast cancer, via both in vitro and in vivo models, with little toxicity to mice. Interestingly, this antimetastatic function is achieved by suppressing the NF-κB signaling pathway, which is important for cancer progression and metastasis, but independent of STING, a previously identified c-di-GMP sensor and NF-κB regulator in mammalian cells. Surprisingly, c-di-GMP inhibits NF-κB activity (p-p65) by directly binding to the proteasome 26S subunit non-ATPase 3 (PSMD3) that we identified as a new TBK1-binding activator, and disrupting the interaction between PSMD3 and TBK1. This PSMD3-TBK1 interaction boosts the phosphorylation and activation of TBK1, representing a noncanonical function of PSMD3 distinct from its established role in proteasomal degradation. Significantly, PSMD3 is highly expressed in malignant and metastatic breast cancers, particularly triple-negative breast cancer. The compelling evidence strongly suggests PSMD3 as a promising target for developing a therapy against metastatic breast cancer. These findings underscore the high potential of c-di-GMP as a safe and effective therapeutic agent for metastatic cancers by targeting the PSMD3-TBK1-NF-κB pathway.

## Introduction

Metastasis, a defining hallmark of cancer, is a highly complex and multistep process through which malignant cancer cells detach from the primary tumor, invade surrounding and distant tissues, and ultimately establish secondary tumors at remote organs.^1,2^ The development of metastatic disease is closely associated with poor clinical outcomes and remains the primary cause of cancer-related mortality worldwide.^3^ Among various cancer types, breast cancer is a major contributor to metastatic burden, with triple-negative breast cancer (TNBC), poses a significant clinical challenge because of its aggressive nature and high recurrence rates. TNBC is characterized by high invasiveness, early relapse, and a strong propensity to metastasize to vital organs, including the lung, liver, bone, and brain.^4,5^ Unlike hormone receptor-positive or HER2-positive breast cancer subtypes, TNBC lacks well-defined molecular targets, severely limiting the availability of effective targeted therapies. Current treatment options largely rely on chemotherapy, which provides limited effects and is frequently accompanied by drug resistance and cancer recurrence.^5^ Given these clinical challenges, identifying novel molecular drivers of metastasis and developing targeted therapeutic strategies are urgently needed to effectively suppress metastatic progression and improve outcomes for patients with advanced breast cancer.

Metastasis relies on a subset of highly aggressive cancer cells that acquire the ability to adapt to dynamic microenvironments, survive detachment and circulation, and successfully colonize in distant organs. Throughout this complex process, metastatic cells must overcome multiple barriers, including immune surveillance, and metabolic stress. Although both the innate and adaptive immune systems play crucial roles in antitumor immune defense, they can also contribute to an immunosuppressive microenvironment that fosters the development of aggressive metastatic tumors.^3,6,7^ 3′,5′-Cyclic diguanylic acid (cyclic di-GMP; c-di-GMP; cdG), a bacterial intracellular signaling molecule, was originally identified as an allosteric activator of cellulose synthase in *Acetobacter xylinum*.^8,9^ While ubiquitous in bacteria and absent in mammals, emerging evidence suggests that c-di-GMP interacts with the innate immune system, modulating host immune responses.^9,10^ Notably, c-di-GMP was found to be a low-affinity ligand for stimulator of interferon genes (STING), a key regulator of the cancer-immunity cycle.^11–14^ STING, a transmembrane protein located in the endoplasmic reticulum, is widely expressed in both immune and nonimmune cells and plays a critical role in innate immune signaling.^11–13,15^ As a STING ligand, c-di-GMP has been extensively studied and employed as a vaccine adjuvant in cancer therapy, overcoming immune suppression and enhancing vaccination efficacy against various cancers, including melanoma and metastatic breast cancer.^10,16^ However, the therapeutic potential of c-di-GMP as a single agent in cancer therapy, especially against metastatic tumors, remains unclear and needs further investigation.

In our attempt to address this remaining issue, we uncovered a previously unrecognized function of c-di-GMP in suppressing cancer metastasis. Surprisingly, our findings reveal that this antimetastatic effect occurs independently of STING in malignant cancer cells, including metastatic breast cancer cells. To elucidate the underlying mechanisms, we performed mass spectrometry analysis of proteins pulled down with c-di-GMP and identified PSMD3 (proteasome 26S subunit, non-ATPase 3; also known as RPN3) as a top c-di-GMP-binding protein candidate. PSMD3 is a component of the 26S proteasome, a critical component of the ubiquitin‒proteasome system, comprising a 20S proteolytically active core particle and two 19S regulatory particles. The 19S particle is further divided into a base subcomplex, containing six ATPases (PSMC1--6) and three non-ATPases (PSMD1, 2, 4), and a lid subcomplex with nine non-ATPases (PSMD3, 6–8, 11–14, and SHFM1).^17,18^ PSMD3 is a relatively understudied component of the proteasome regulatory machinery. Emerging evidence has begun to implicate the regulatory effects of PSMD3 in the multiple human malignancies, including breast cancer. PSMD3 has been shown to influence tumor progression by modulating diverse cancer-associated factors.^19–22^ These observations imply that PSMD3 may play a role in cancer progression beyond its canonical involvement in proteasome-mediated protein degradation and may serve as a potential molecular target for developing an anticancer therapy. Although PSMD3 is widely expressed across mammalian tissues and cell types, the proteasome-independent functions of PSMD3, as well as the molecular mechanisms by which it contributes to oncogenic signaling, remain largely unexplored. Elucidating the noncanonical function of PSMD3 would provide new insights into tumor biology and uncover previously unrecognized therapeutic opportunities.

Our further analyses described below reveal that PSMD3 is a novel molecular target of c-di-GMP. Interestingly, we found that c-di-GMP suppresses metastasis by inhibiting NF-κB activation (phosphorylation of p65 at Ser536). This inhibition was achieved by interrupting the interaction between PSMD3 and TBK1, consequently attenuating TBK1 activation and downstream signaling. Thus, our study not only reveals a novel antimetastatic activity of c-di-GMP, but also identifies the noncanonical function of PSMD3 as a novel regulator of TBK1 that directly binds to its coiled-coil domain and a new therapeutic target of this small molecule. It also highlights the potential of c-di-GMP as a powerful antimetastatic agent for clinical therapy against metastatic breast cancer and possibly other metastatic malignancies.

## Results

### c-di-GMP inhibits cancer metastasis

**Fig. 1:**
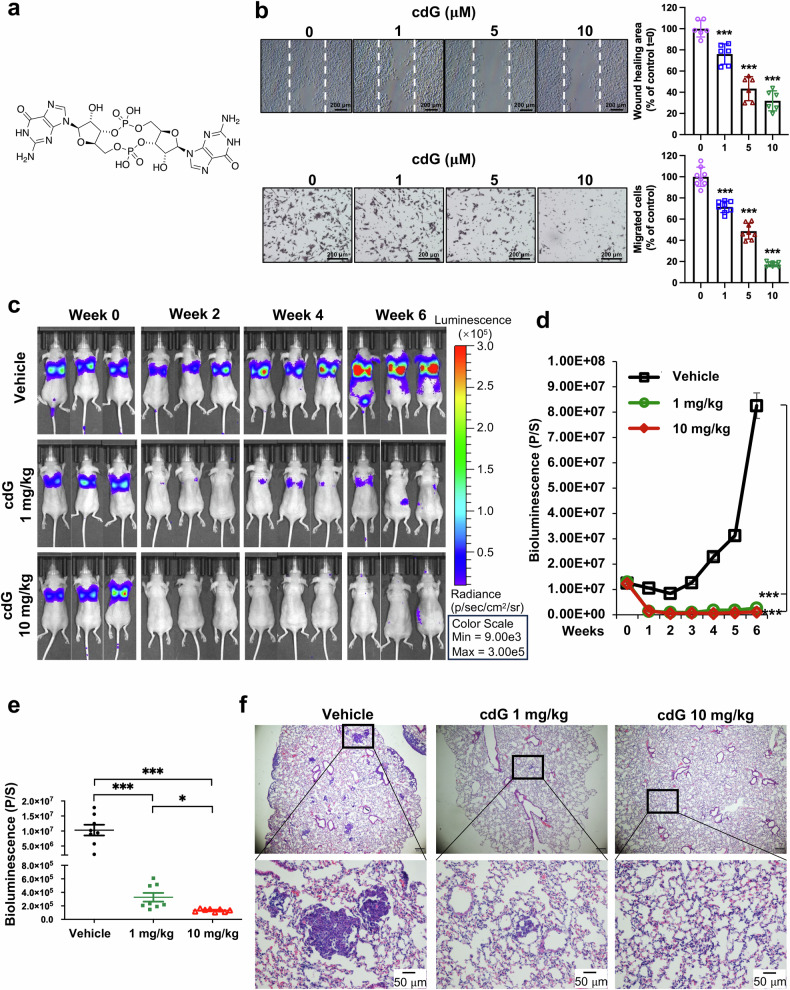
c-di-GMP inhibits human cancer metastasis in nude mice. **a** Chemical structure of c-di-GMP (cdG, 3′,5′-cyclic diguanylic acid or 3′,5′-cyclic dimeric guanosine monophosphate). **b** Representative images of the wound-healing assay (upper) and transwell migration assay (lower) performed on MDA-MB-231 cells treated with the indicated doses of c-di-GMP for 15–17 h. Migrated cells were quantified manually. ***P < 0.001 compared with the control group (*n* = 6–8 random fields from 3 separate wells or inserts per condition). The data are presented as the means ± SDs. Scale bar, 200 μm. **c** Representative bioluminescence images of breast cancer cell metastases. MDA-MB-231-luc cells were injected intravenously into female nude mice. PBS or c-di-GMP was administered intraperitoneally on day 0 and subsequently every other day. Lung metastases were monitored weekly via an in vivo imaging system. **d** Quantification of bioluminescence (luciferase flux; p/s = photons/second) for lung metastases. *n* = 8 mice per group. **e** Mean bioluminescence at the end of the indicated treatments. Each dot represents an individual mouse (*n* = 8 per group). The data are presented as the means ± SEMs. *P < 0.05; ***P < 0.001 (one-way ANOVA followed by Bonferroni’s multiple comparison test). **f** H&E staining of lung sections from the indicated groups harvested on day 42. Scale bar = 50 μm as indicated

As a recently reported ligand of STING,^14^ c-di-GMP (Fig. 1a) is widely used as a vaccine adjuvant in cancer therapy to overcome immune suppression and improve vaccine efficacy against different cancer types, including metastatic breast cancer.^10^ To determine whether c-di-GMP exerts direct effects on cancer cells, we tested its impact on cell viability in various human cancer cell lines, including breast cancer cells (MDA-MB-231, MCF-7 and BT-549), pancreatic cancer cells (PANC-1 and AsPC-1), colon cancer LoVo cells, and NSCLC H1299 cells (Supplementary Fig. 1). c-di-GMP had little to moderate effects on the growth of these cancer cells, with IC_50_ values mostly exceeding 100 μM for up to 3 days. However, even at a concentration of 1 μM, c-di-GMP significantly inhibited cancer cell migration, as detected by wound healing (Fig. 1b, MDA-MB-231 cells; Supplementary Fig. 2a–c, BT-549, PANC-1 and LoVo cells) and transwell assays (Fig. 1b).

To further determine the biological effect of c-di-GMP on cancer metastasis, we utilized a lung metastasis mouse model by injecting highly metastatic luciferase-labeled MDA-MB-231 human breast cancer cells via the tail vein and tracking the metastatic tumor burden via IVIS bioluminescence imaging. The mice received an intraperitoneal injection of c-di-GMP (1 mg/kg or 10 mg/kg) every other day. Remarkably, c-di-GMP suppressed lung metastasis even at the lower dose (1 mg/kg), with nearly complete inhibition at 10 mg/kg, as evidenced by bioluminescence imaging, which revealed that almost no metastatic cells were detected in the animals’ lungs starting at week 2 postadministration (Fig. 1c–e). Histological analysis (H&E staining) of the lung tissues confirmed a reduction in the number of tumor nodules following c-di-GMP treatment (Fig. 1f). Moreover, c-di-GMP treatment was well tolerated for six weeks, with no significant changes in body weight or evidence of tissue injury (Supplementary Fig. 3a, b). Similar antimetastatic effects were observed in an independent B16-F10 metastatic melanoma model (Supplementary Fig. 4a–d). These findings demonstrate that c-di-GMP possesses antimetastatic activity even at low doses without causing detectable adverse effects.

### c-di-GMP-specific antimetastatic activity is STING independent

**Fig. 2:**
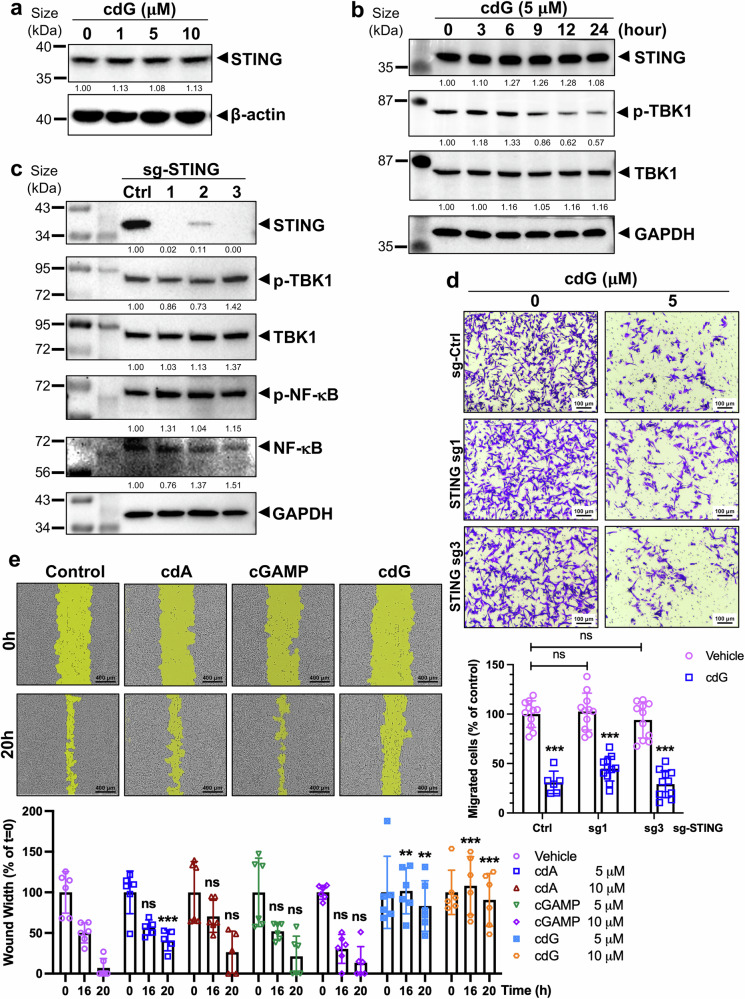
The antimetastatic effect of c-di-GMP is independent of STING. **a** MDA-MB-231 cells were treated with the indicated doses of c-di-GMP for 24 h, and total STING levels were assessed by WB analysis. The numbers underneath the bands represent the normalized density quantified by densitometry via ImageJ. **b** MDA-MB-231 cells were treated with 5 μM c-di-GMP for the indicated time points (3–24 h), and total STING levels and downstream protein expression were assessed via WB analysis. **c** Genetic knockout of STING in MDA-MB-231 cells was achieved via CRISPR-Cas9. Several MDA-MB-231 clones stably expressing Cas9 and either control sgRNA (sg-Ctrl) or three different sgRNAs targeting STING were isolated. Total STING levels and downstream protein expression were assessed via WB analysis. **d** Metastasis was analyzed via a transwell migration assay with or without 5 μM c-di-GMP for 16 h in STING KO clones. Representative images are shown (upper), and migrated cells were manually quantified (lower). *n* = 6‒11 random fields from 3 separate inserts per condition. The data are presented as the means ± SDs. ***P < 0.001 compared with the control group; ns not significant. Scale bar, 100 μm **e** The migration of MDA-MB-231 cells following treatment with different concentrations of STING agonists (c-di-AMP, 2′3′-cGAMP or c-di-GMP) was evaluated via a wound-healing scratch assay at the indicated time points. An IncuCyte WoundMaker kit was used, and images were captured via the IncuCyte Zoom system. Representative images showing the 5 μM treatment at 20 h. Wound width was analyzed via IncuCyte software (*n* = 6). Scale bar, 400 μm. The results were quantified and are presented as the means ± SDs; ***P < 0.001; **P < 0.01; ns not significant

STING is widely expressed in both immune and nonimmune cells, including cancer cells.^11,15^ However, its expression is often suppressed or reduced in many cancers, particularly in more advanced stages.^23^ To investigate whether the antimetastatic effects of c-di-GMP are dependent on STING signaling, we examined STING protein levels in a panel of cancer cell lines. Notably, highly metastatic breast cancer cell lines (MDA-MB-231, BT-549 and Hs578T) presented low STING expression, with HEK293T cells serving as a negative control (Supplementary Fig. 5a). Because the cGAS-STING pathway has been studied predominantly for its role in mediating innate immune responses during infection and other cellular stresses,^12,24^ we sought to determine whether the antimetastatic effects of c-di-GMP are dependent upon STING signaling. To do so, we treated various cancer cell lines with increasing concentrations (1, 5, and 10 μM) of c-di-GMP for 24 h. First, the c-di-GMP treatments did not affect STING protein levels in MDA-MB-231 cells or in other cancer cells with relatively high STING levels, such as H1299, Calu-1 and SW1990 cells (Fig. 2a and Supplementary Fig. 5b, c). Moreover, as shown in Fig. 2b (Supplementary Fig. 5d), treatment with 5 μM c-di-GMP for different durations (0–24 h) did not alter the STING protein level. However, c-di-GMP markedly reduced the phosphorylation of TBK1 at Ser172 (p-TBK1) beginning at 9 h and lasting up to 24 h in a time-dependent fashion, as further described in the following section. In support of this result, STING knockout neither affected the levels of some proteins in the TBK1-NF-κB pathway nor altered the phosphorylation levels of TBK1 and NF-κB (p65) in MDA-MB-231 cells (Fig. 2c). Moreover, depletion of STING via siRNAs (Supplementary Fig. 5f, right panel) or CRISPR (Fig. 2c, top panel) in MDA-MB-231 cells, as detected by Western blot (WB) analysis, had no effect on cell migration (Fig. 2d, left column and Supplementary Fig. 5f, left panels). However, c-di-GMP still significantly inhibited cell migration, even in the absence of STING (Fig. 2d, right column). Moreover, as shown in Supplementary Fig. 5e, c-di-GMP treatment did not affect total or phosphorylated IKKε levels but did reduce p-TBK1 levels in a dose-dependent manner, which is consistent with the results shown in Fig. 2b. These results indicate that the antimetastatic effects of c-di-GMP occur independently of STING signaling in cancer cells.

In addition to c-di-GMP, STING also binds to other cyclic dinucleotides (CDNs), including both endogenous 2′3’-cGAMP (cGAMP) and exogenous c-di-AMP (cdA) or 3′3’-cGAMP from bacteria. To investigate whether other CDNs also influence the metastatic potential of malignant cancer cells, we tested their effects in our system. Surprisingly, among the CDNs known to activate the STING pathway in immune responses, c-di-GMP exhibited the strongest and most consistent inhibitory effects on metastasis among these CDNs (Fig. 2e). These findings indicate that the antimetastatic effects of c-di-GMP are STING independent and specific to c-di-GMP rather than being a general property of CDNs.

### c-di-GMP inhibits the NF-κB signaling pathway

**Fig. 3:**
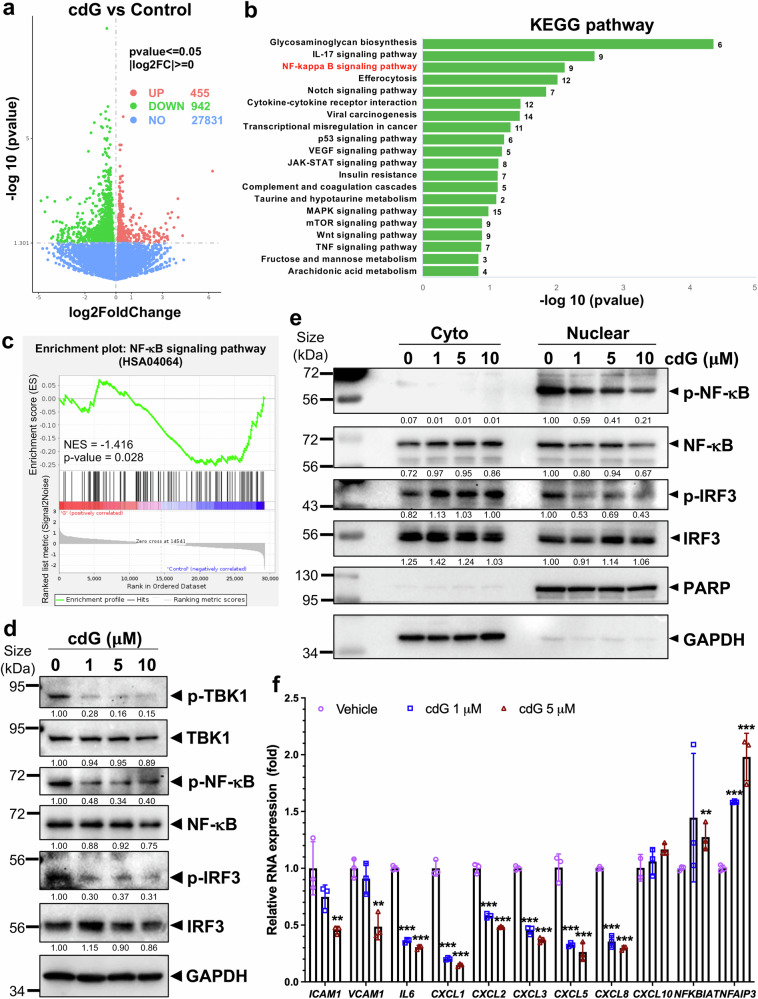
c-di-GMP suppresses the NF-κB signaling pathway. **a** Volcano plot of overall differentially expressed genes (DEGs) between the c-di-GMP treatment and control groups identified via RNA-seq. *p* value < 0.05; |log2FoldChange| > 0. **b** KEGG enrichment analysis of genes downregulated in MDA-MB-231 cells following c-di-GMP treatment. The top 20 signaling pathways are shown. **c** Gene set enrichment analysis (GSEA) of the NF-κB signaling pathway between the c-di-GMP treatment group and the control group (NES = −1.416; *p* value = 0.028). **d** MDA-MB-231 cells were treated with increasing concentrations of c-di-GMP for 24 h, and whole-cell lysates were analyzed by Western blotting. **e** c-di-GMP inhibits the nuclear localization of phosphorylated NF-κB (p-NF-κB Ser536). MDA-MB-231 cells were treated with different concentrations of c-di-GMP for 24 h, stimulated with TNFα (1 nM) for 1 h, and then subjected to cell fractionation. GAPDH was used as a cytoplasmic marker, and PARP was used as a nuclear marker. **f** The mRNA expression levels of NF-κB target genes were assessed via RT‒qPCR following c-di-GMP treatment for 24 h. Data were quantified and are presented as a graph with the means ± SDs, *n* = 3. *p < 0.05, **p < 0.01, ***p < 0.001 by two-tailed *t* test

To elucidate the molecular mechanism(s) underlying c-di-GMP-mediated metastasis inhibition, we performed RNA-sequencing (RNA-seq) analysis of MDA-MB-231 cells treated with 5 μM c-di-GMP for 24 h (*n* = 4). Differentially expressed genes (DEGs) were identified via the DESeq2 R package (1.20.0) with thresholds of absolute log_2_-fold change > 0 and *p* value < 0.05. A total of 1397 DEGs were detected, including 455 upregulated and 942 downregulated genes (Fig. 3a). The 50 most significantly upregulated and downregulated DEGs after c-di-GMP treatment are shown in a heatmap (Supplementary Fig. 6b). KEGG pathway enrichment analysis from two independent RNA-seq analyses consistently revealed significant downregulation of genes involved in the NF-κB, TNFα and JAK-STAT signaling pathways in the c-di-GMP-treated cells compared with the untreated controls, with NF-κB signaling being the most suppressed pathway among the three (Fig. 3b, Supplementary Fig. 6a and Supplementary Table 5). Gene set enrichment analysis (GSEA) further revealed that the NF-κB signaling pathway was significantly downregulated upon c-di-GMP treatment (NES = −1.416; *p* value = 0.028) (Fig. 3c), along with a similar trend for the TNFα signaling pathway (NES = −1.328; *p* value = 0.053) (Supplementary Fig. 6c).

On the one hand, NF-κB proteins regulate the expression of hundreds of genes involved in inflammation, immunity, proliferation, and cell death, playing a key role in maintaining cellular homeostasis in response to various stimuli, such as cytokines, TNFα, and interleukin 1 beta (IL-1β).^25^ On the other hand, NF-κB pathway activation also induces the expression of inflammatory cytokines, chemokines, and their receptors, establishing positive feedback regulation of the immune response.^26^ This pathway has been shown to be crucial for cancer cell metastasis.^27,28^ Thus, we investigated whether c-di-GMP affects NF-κB signaling in cancer cells. Interestingly, c-di-GMP drastically reduced NF-κB phosphorylation at Ser536 (p-p65) (Fig. 3d and Supplementary Fig. 6d). Notably, the phosphorylation of p65 at serine 536 has been shown to be essential for its nuclear translocation.^29^ Under physiological conditions, the NF-κB subunit p65 is largely retained in the cytoplasm of unstimulated cells and is minimally detected in the nucleus. TNFα stimulation induces rapid phosphorylation of p65 and its subsequent nuclear translocation, events that serve as indicators of NF-κB pathway activation.^30^ To study the effect of c-di-GMP on p65 nuclear translocation, we pretreated cells with c-di-GMP followed by 1 nM TNFα stimulation for 1 h prior to cell fractionation. As shown in Fig. 3e, c-di-GMP treatment reduced the nuclear import of p-p65 upon TNFα activation in a dose-dependent fashion. This treatment also reduced p-TBK1 and p-IRF3 levels as well as the nuclear import of p-IRF3 (Fig. 3d, e and Supplementary Fig. 6d). Quantitative real-time PCR (Q-PCR) confirmed the RNA-seq findings, demonstrating the downregulation of NF-κB target genes,^31,32^ including key cytokines and the chemokines *CXCL1*, *CXCL2*, *CXCL3*, *CXCL5*, *CXCL8*, *IL6*, *ICAM1* and *VCAM1*, and the upregulation of NF-κB negative feedback regulators, such as *NFKBIA* and *TNFAIP3*, following c-di-GMP treatment (Fig. 3f; primer sequences are presented in Supplementary Table 3). Together with the findings in Fig. 2, these results indicate that c-di-GMP inhibits metastasis by suppressing a STING-independent NF-κB pathway, leading to the downregulation of proinflammatory cytokines and chemokines and disrupting the feedback loop that facilitates cancer cell migration and metastasis.

### c-di-GMP binds directly to a newly identified target, PSMD3

**Fig. 4:**
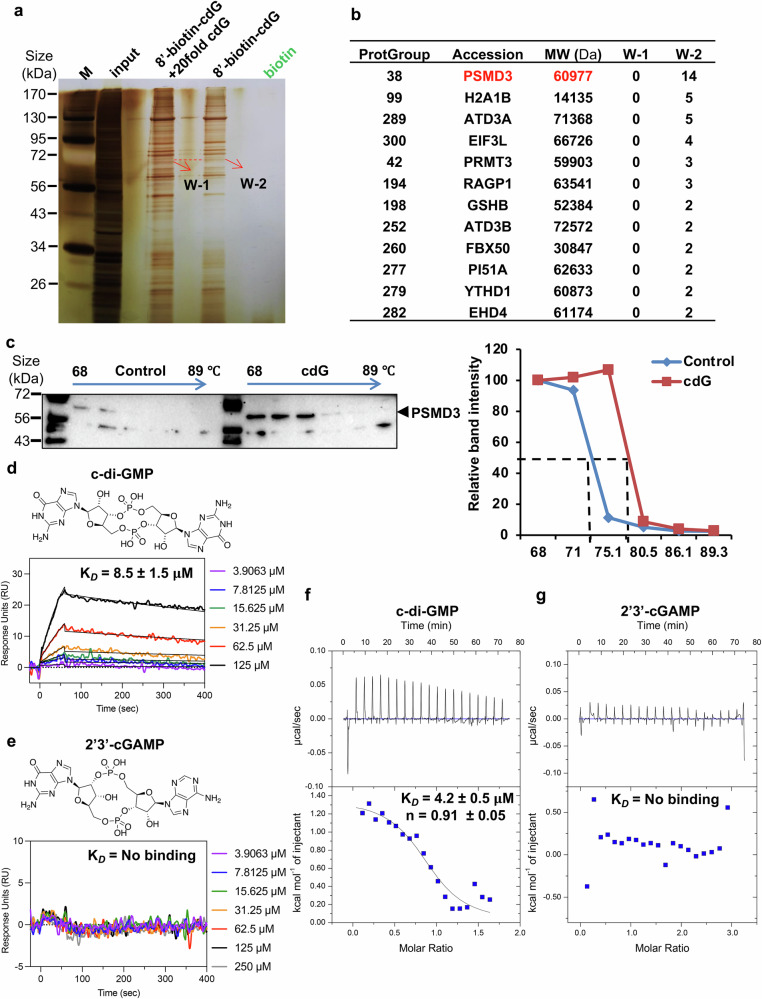
c-di-GMP directly binds to PSMD3 in breast cancer cells and in vitro. **a** MDA-MB-231 cell lysates were incubated with biotin-c-di-GMP or biotin alone in the absence or presence of a 20-fold excess of unlabeled c-di-GMP, followed by pull-down using streptavidin-agarose. The precipitates were resolved via SDS‒PAGE and visualized via silver staining. The arrows indicate bands excised for LC–MS-MS analysis (W-2, the band specifically pulled down by biotin-c-di-GMP; W-1, the control band; binding candidates competed out by excess unlabeled c-di-GMP). **b** Table listing protein candidates directly targeted by c-di-GMP identified in the W-2 group with at least two unique peptides via LC‒MS/MS. **c** Cellular thermal shift assay (CETSA). MDA-MB-231 cell lysates were incubated with or without c-di-GMP, followed by exposure to different temperatures. PSMD3 protein levels were analyzed by Western blotting (left). The expression ratio of PSMD3 (normalized to its expression at the lowest temperature) was quantified via ImageJ (right). **d** Chemical structure of c-di-GMP (upper), which is composed of two guanine nucleotides linked via 3′–5′ phosphodiester bonds. SPR sensorgrams showing the concentration-dependent binding of c-di-GMP to immobilized recombinant PSMD3 E61–S460. A six-point, twofold dilution series (3.9–125 μM) was used. The colored traces represent experimental data, and the black lines indicate global fits to a 1:1 binding model, yielding an apparent equilibrium dissociation constant (K_*d*_ = 8.5 ± 1.5 μM). **e** Chemical structure of 2′3′-cGAMP (upper), composed of guanine and adenine nucleotides linked via mixed 2′–5′ and 3′–5′ phosphodiester bonds. SPR sensorgrams showing no detectable binding between PSMD3 and 2′3′-cGAMP under identical experimental conditions to those for panel (**d**). No measurable association or dissociation was observed across a seven-point, twofold dilution series of analyte concentrations (3.9–250 μM), indicating the absence of a detectable interaction. **f** ITC profile of PSMD3 titrated with c-di-GMP at 25 °C. The raw thermogram (upper) and integrated heat (lower) show a saturating exothermic isotherm fit to a one-site model (K_*d*_ = 4.2 ± 0.5 μM; *n* = 0.91 ± 0.05; Δ*H* ≈ –1.3 kcal·mol⁻¹; *TΔS* ≈ +8.7 kcal·mol⁻¹). **g** ITC analysis of PSMD3 titrated with 2′3′-cGAMP under the same buffer and temperature conditions as (**f**) showing no significant heat exchange, which is consistent with a lack of binding under saturating conditions

To identify the direct molecular target of c-di-GMP, we performed a biotin-c-di-GMP pull-down assay followed by mass spectrometric analysis in MDA-MB-231 cells, with free biotin used as a control. First, the antimetastatic effect of biotinylated c-di-GMP (8-biotin-11-c-di-GMP; Supplementary Fig. 7b) was confirmed to be comparable to that of c-di-GMP, as demonstrated by a transwell assay (Supplementary Fig. 7a). The precipitated proteins were analyzed via gel electrophoresis and silver staining. A distinct ~65 kDa protein band was precipitated by biotin-c-di-GMP (W-2), and its presence was competitively reduced by a higher concentration of unlabeled c-di-GMP (W-1) (Fig. 4a, enlarged view shown in Supplementary Fig. 7c), suggesting that this band represents a c-di-GMP-binding protein. The gel pieces corresponding to the regions indicated by the red frames (W-1 and W-2; Supplementary Fig. 7c, right) were excised and then subjected to mass spectrometric analysis to identify c-di-GMP-binding proteins. Unexpectedly, the top candidate identified was PSMD3 (Fig. 4b and Supplementary Table 4), an ~60 kDa protein subunit of the 19S regulatory particle of the 26S proteasome.^17,18^ The c-di-GMP-PSMD3 interaction was validated via a cellular thermal shift assay (CETSA) followed by WB analysis, where c-di-GMP markedly increased the thermal stability of PSMD3 compared with that of the control group in MDA-MB-231 cells (Fig. 4c).

To further confirm the direct binding of c-di-GMP to PSMD3, we first purified a truncated, soluble version of PSMD3 expressed in *E. coli* (Supplementary Fig. 8a). As described in the Methods, the recombinant PSMD3 protein was highly purified with more than 98% purity, as confirmed by a Coomassie blue-stained SDS‒PAGE gel (Supplementary Fig. 8a). Next, we examined the binding affinity of c-di-GMP for PSMD3 via surface plasmon resonance (SPR) assays. The purified PSMD3 protein was immobilized on a CM5 SPR chip, and different concentrations of c-di-GMP and 2’3’-cGAMP were tested. The resulting sensorgrams clearly displayed concentration-dependent binding to c-di-GMP, with a binding affinity in the low micromolar range (Fig. 4d). The reported kinetic rate constants (k_on_ and k_off_) were obtained from global fitting of the concentration series to a 1:1 Langmuir binding model. The calculated parameters (k_on_ = 87.9 ± 19.7 M⁻¹ s⁻¹, k_off_ = (7.55 ± 2.64) × 10⁻⁴ s⁻¹) correspond to an equilibrium dissociation constant of (8.51 ± 1.53) × 10⁻⁶ M (K_*d*_ ≈ 8.5 μM), which is consistent across three independent replicates. This affinity falls within the physiological range observed for other established c-di-GMP-binding proteins and is comparable to its binding affinity for STING (K_*d*_ 2.4–14.5 μM).^33,34^ In contrast, 2’3’-cGAMP produced no measurable binding signal even at higher analyte concentrations (Fig. 4e), confirming the specificity of PSMD3 for c-di-GMP.

To independently confirm these kinetic results and dissect the underlying thermodynamic parameters, we next performed isothermal titration calorimetry (ITC) as a saturating, label-free technique to determine the binding affinity of PSMD3–ligand interactions. Titration of c-di-GMP into PSMD3 produced a clear, saturating exothermic binding isotherm that fit well with a one-site model, yielding a dissociation constant (K_*d*_ = 4.2 ± 0.5 μM) and a stoichiometry of approximately one ligand per protein molecule (*n* = 0.91 ± 0.05) (Fig. 4f). The interaction was characterized by a modestly favorable enthalpy (ΔH ≈ –1.3 kcal·mol⁻¹) and a dominant entropy contribution (TΔS ≈ +8.7 kcal·mol⁻¹), which is consistent with specific hydrogen bonding and hydrophobic desolvation. In contrast, 2′3′-cGAMP produced no detectable binding response even when titrated at two analyte concentrations under identical buffer and temperature conditions (Fig. 4g), confirming the selective recognition of c-di-GMP.

To further assess specificity, we used ITC to test GRP78, a cancer-associated nucleotide-binding chaperone, as a negative control. GRP78 was purified via immobilized metal affinity chromatography and size-exclusion chromatography, yielding a highly pure, single band at the expected molecular weight on a Coomassie blue-stained SDS‒PAGE gel (Supplementary Fig. 8b). GRP78 showed no detectable heat signal when titrated with c-di-GMP under identical conditions (Supplementary Fig. 8c), indicating that the PSMD3–c-di-GMP interaction is not due to nonspecific nucleotide affinity. Together, these orthogonal biophysical measurements establish PSMD3 as a selective c-di-GMP-binding protein that is distinct from the canonical metazoan cyclic dinucleotide sensors that recognize 2’3’-cGAMP. This observation is also consistent with our earlier findings (Fig. 2e), which revealed that only c-di-GMP, but not 2’3’-cGAMP, has antimetastatic effects.

### PSMD3 is a novel TBK1-binding protein

**Fig. 5:**
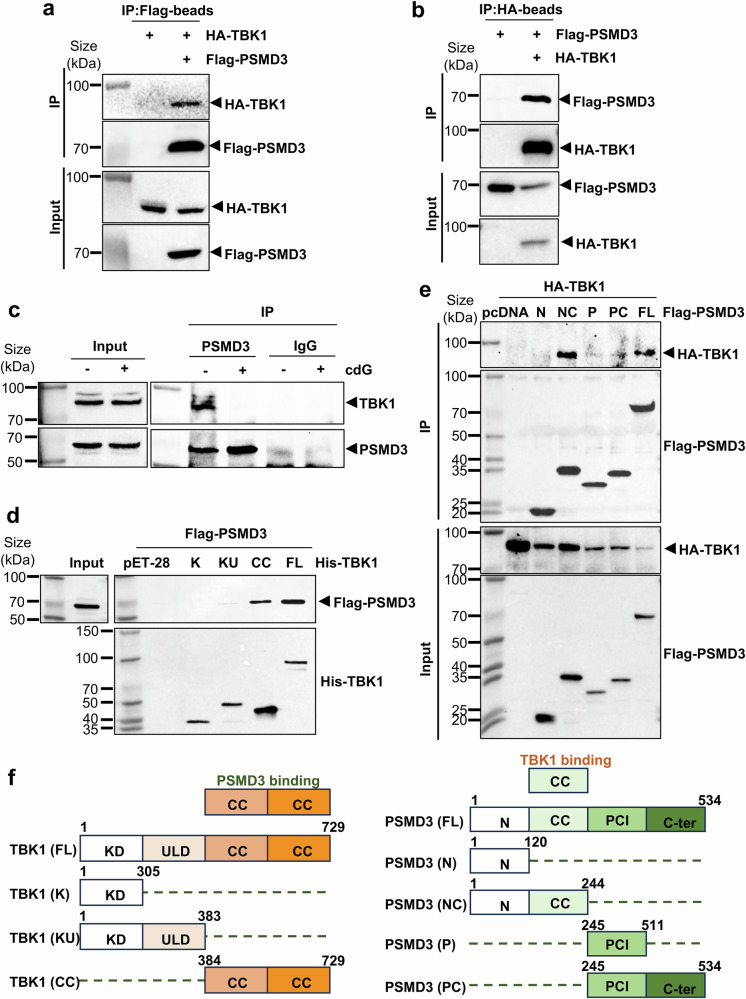
PSMD3 and TBK1 bind to each other via their coiled coil domains. **a** HEK293T cells were transfected with HA-TBK1 and Flag-PSMD3 plasmids as indicated. Anti-Flag beads were used for the co-IP assay. **b** HEK293T cells were transfected with Flag-PSMD3 and HA-TBK1 plasmids as indicated, followed by co-IP using anti-HA beads. **c** Endogenous PSMD3-TBK1 complexes in MDA-MB-231 cells were detected by co-IP using a PSMD3 antibody with IgG as a negative control. Treatment with c-di-GMP (5 μM, 24 h) disrupted the endogenous interaction between PSMD3 and TBK1 in MDA-MB-231 cells. **d** His-tagged TBK1 fragments and full-length fusion proteins were expressed in *E. coli* and purified via HisPur™ Ni-NTA Resin. Equal amounts of lysates from Flag-PSMD3-transfected HEK293T cells were incubated with these beads. Binding between TBK1 and PSMD3 was analyzed by WB using an anti-FLAG antibody. Equal loading of His-tagged proteins was confirmed by WB analysis with an anti-His antibody. **e** HA-tagged TBK1 and Flag-tagged PSMD3 full-length and truncated plasmids were cotransfected into HEK293T cells as indicated, followed by a co-IP assay using anti-Flag beads. **f** Left, schematic representation of the PSMD3 binding region on TBK1. FL full length, KD kinase domain, ULD ubiquitin-like domain, CC coiled-coil domain, Right, schematic representation of the TBK1-binding region within the PSMD3 domain structure. FL full length, N,N-terminal fragment (unstructured region containing potential regulatory motifs), CC connector region (coiled-coil–like helical segment), PCI proteasome component domain (core functional domain), C-ter C-terminus

Our findings that c-di-GMP inhibits the TBK1-NF-κB signaling pathway (Fig. 3) and directly binds to PSMD3 (Fig. 4) suggest that PSMD3 plays a role in TBK1-NF-κB signaling. To explore the underlying mechanism, we first examined the possible interaction between PSMD3 and TBK1. As a result, we observed that PSMD3 coimmunoprecipitated with ectopic TBK1, a well-established NF-κB kinase,^35^ in HEK293T cells cotransfected with Flag-PSMD3 and HA-TBK1 plasmids with empty vector controls included in reciprocal co-IP experiments (Fig. 5a, b). The endogenous TBK1-PSMD3 complex was also detected in MDA-MB-231 breast cancer cells via co-IP-WB analysis (Fig. 5c). Interestingly, c-di-GMP treatment markedly reduced the formation of the PSMD3-TBK1 complex in the cells (Fig. 5c and Supplementary Fig. 9a). This ability to bind to TBK1 was specific to PSMD3, as endogenous TBK1 was not pulled down with the other two 19S subunits, PSMC4 and PSMD4 (Supplementary Fig. 9c, d). Together, these results demonstrate that PSMD3 is a novel TBK1-binding protein, as further verified below.

To map the direct binding domains between PSMD3 and TBK1, we expressed and purified His-tagged TBK1 fragments and full-length fusion proteins from *E. coli* and incubated them with lysates containing full-length Flag-PSMD3. As shown in Fig. 5d and Supplementary Fig. 9b, full-length PSMD3 was pulled down by full-length TBK1 and its coiled-coil (CC) domain (amino acids (aa) 384–729) but not by other fragments. TBK1 contains a kinase domain (KD, aa 1–305), a ubiquitin-like domain (ULD, aa 306–383) and coiled-coil domains (CCs, aa 384–729) (Fig. 5f). To map the TBK1-binding domain(s) of PSMD3, we cointroduced full-length HA-tagged TBK1 with Flag-tagged PSMD3 or its truncated constructs into HEK293T cells as indicated and conducted a co-IP-WB assay. As shown in Fig. 5e, the connector region of PSMD3 (a coiled-coil–like helical segment, aa 121–244) was critical for TBK1 binding. These results demonstrate that PSMD3, via its coiled-coil connector region, directly interacts with the coiled-coil domain of TBK1 (Fig. 5f).

### c-di-GMP suppresses cancer cell migration by targeting PSMD3

**Fig. 6:**
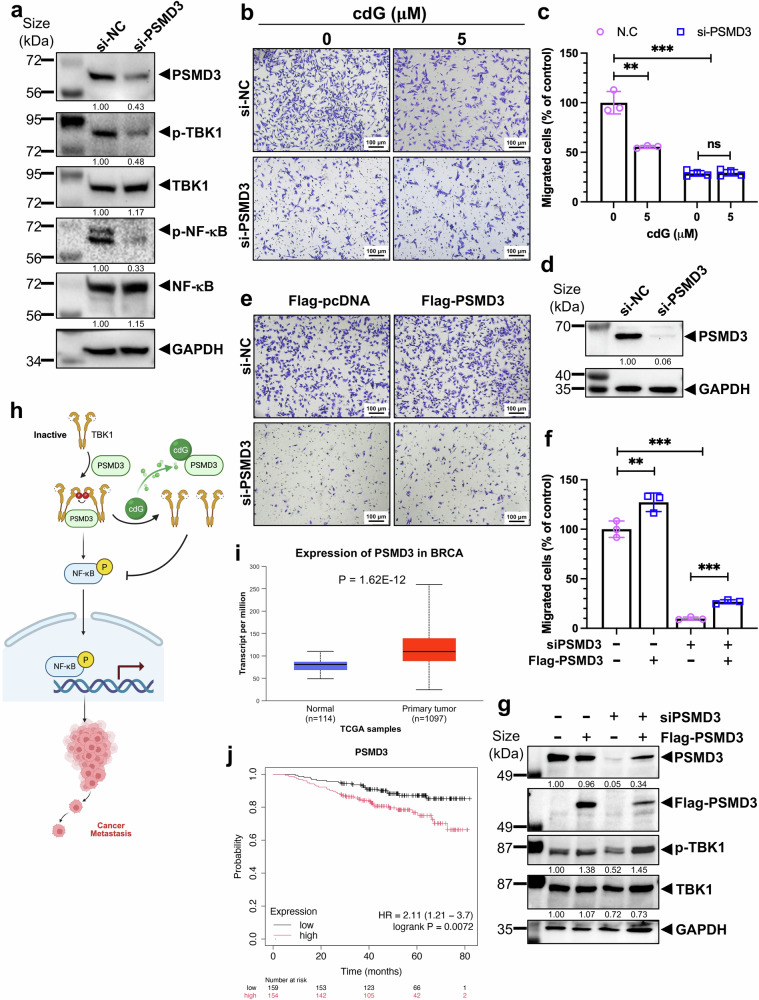
PSMD3 is required for cancer cell migration and is highly expressed in aggressive and late-stage breast cancers. **a** WB analysis of the effect of PSMD3 knockdown on TBK1-NF-κB activation in MDA-MB-231 cells. **b** PSMD3 knockdown in MDA-MB-231 cells was achieved via siRNA. Cell migration was analyzed via a transwell migration assay with or without 5 μM c-di-GMP treatment for 16 h. Representative images are shown. Scale bar, 100 μm. **c** Migrated cells were quantified via ImageJ software and normalized to the control. ***P < 0.001, **P < 0.01 compared with the control group; ns not significant (*n* = 3‒4 separate inserts per condition). The data are presented as the means ± SDs. **d** The efficiency of PSMD3 knockdown was confirmed by immunoblotting. **e** MDA-MB-231 cells were transfected with the indicated siRNAs and/or Flag-PSMD3 for 72 h, followed by a transwell migration assay. Representative images are shown. Scale bar, 100 μm. **f** Migrated cells were quantified by ImageJ software and normalized to the control. ***P < 0.001, **P < 0.01 compared with the control group (*n* = 3 separate inserts per condition). The data are presented as the means ± SDs. **g** The efficiency of PSMD3 knockdown and overexpression in the same batch of transwell assays (**e**) was confirmed by immunoblotting, and the effects on TBK1 activation were analyzed. **h** Schematic illustration of the proposed mechanism by which c-di-GMP suppresses the PSMD3-TBK1-NF-κB pathway. **i** Relative mRNA expression of *PSMD3* in normal (*n* = 114) and breast cancer (*n* = 1097) tissues from the UALCAN Breast Invasive Carcinoma Cohort (*p* = 1.62E−12). **j** Kaplan–Meier analysis of PSMD3 expression and overall survival in an untreated breast cancer cohort (*n* = 313) from the Kaplan‒Meier plotter database. The *P* value was calculated via the log-rank test

Given that PSMD3 interacts with TBK1 and serves as a direct target of c-di-GMP, we speculated that PSMD3 functions as a critical regulatory component of the TBK1-NF-κB signaling pathway and that c-di-GMP inhibits cancer cell migration by targeting PSMD3. TBK1 activation involves its dimerization and subsequent phosphorylation, which can occur through trans-autophosphorylation. In its inactive conformation, TBK1 adopts an architecture in which its two dimeric kinase domains are orchestrated on opposing surfaces, a spatial arrangement that prevents cisautophosphorylation and maintains the kinase in an inactive state. Activation of TBK1 is therefore thought to depend on transautophosphorylation that is facilitated by the formation of higher-order oligomerization of TBK1 dimers that then associate with other TBK1 dimers, bringing their active sites into proximity to phosphorylate serine 172 on the adjacent TBK1 partner.^36^ Since PSMD3 interacts with TBK1 within its α-helical scaffold dimerization domain (coiled-coil region; Fig. 5d–f), which could mediate the formation of the compact TBK1 dimer essential for activation, PSMD3 binding to TBK1 could promote the recruitment and close association of TBK1 dimers, increasing the local concentration of TBK1 and enhancing interdimer interactions that facilitate transautophosphorylation and activation. Hence, we first determined whether PSMD3 is required for the activation of TBK1-NF-κB signaling by knocking down endogenous PSMD3 and analyzing several key components of the pathway in MDA-MB-231 cells. Interestingly and surprisingly, PSMD3 knockdown led to a significant reduction in p-TBK1 (Ser172) and p-NF-κB (p-p65 Ser536) protein levels (Fig. 6a and Supplementary Fig. 10a). However, knockdown of PSMD3 did not affect the protein level of c-Myc (Supplementary Fig. 10b), a short living and proteasome-degraded oncoprotein with an approximately 15-minute half-life.^37^ These results suggest that PSMD3 regulation of TBK1 must be independent of the 19S proteasome lid. Together, these results identify PSMD3 as a novel regulator of the TBK1-NF-κB pathway, acting independently of its canonical role in the proteasome, and indicate that c-di-GMP binds to PSMD3 and inhibits its function by interrupting its interaction with and activating TBK1, thereby suppressing NF-κB signaling.

To determine whether PSMD3 is required for the c-di-GMP-mediated inhibition of breast cancer cell migration, we conducted a transwell assay in MDA-MB-231 cells with PSMD3 depletion. As shown in Fig. 6b–d, PSMD3 knockdown significantly reduced cancer cell migration. Remarkably, depletion of PSMD3 attenuated the inhibitory effect of c-di-GMP on cancer cell migration, as there was no significant difference in the number of migrated cells between the c-di-GMP-treated and untreated groups in the absence of PSMD3 (Fig. 6c). To exclude the off-target effects of PSMD3 knockdown, we performed a rescue assay in which ectopic expression of PSMD3 significantly reversed the inhibition of migration caused by PSMD3 depletion in a transwell assay (Fig. 6e, f). Technically and practically, the efficiency of Flag-PSMD3 overexpression in MDA-MB-231 cells was much lower when PSMD3-specific siRNA was also expressed in the cells than when it was knocked down with siRNA alone; therefore, only a small proportion of the cells successfully overexpressed PSMD3 (Fig. 6g). Nonetheless, these cells exhibited significant restoration of migratory ability (Fig. 6e, f). These findings demonstrate that c-di-GMP inhibits cancer cell migration by disrupting the interaction between PSMD3 and TBK1, which is required for TBK1 activation, and the sustained activation of NF-κB signaling, which promotes cancer cell migration (Fig. 6h).

To further explore the clinical relevance of PSMD3 as a c-di-GMP target in breast cancer metastasis, we analyzed publicly available gene expression datasets. Using UALCAN to access TCGA Breast Invasive Carcinoma data, we observed that *PSMD3* mRNA levels were significantly elevated in tumor tissues compared with normal tissues (Fig. 6i and Supplementary Fig. 11b). A similar trend was confirmed in the GEPIA database in BRCA patients (Supplementary Fig. 11a). Consistently, higher *PSMD3* levels were correlated with poorer survival outcomes in breast cancer patients (Fig. 6j and Supplementary Fig. 11c). These findings suggest that elevated PSMD3 expression is associated with reduced survival rates, highlighting its potential as both a prognostic biomarker and a therapeutic target in breast cancer, especially invasive disease.

### c-di-GMP inhibits lung metastasis of mouse orthotopic mammary tumors

**Fig. 7:**
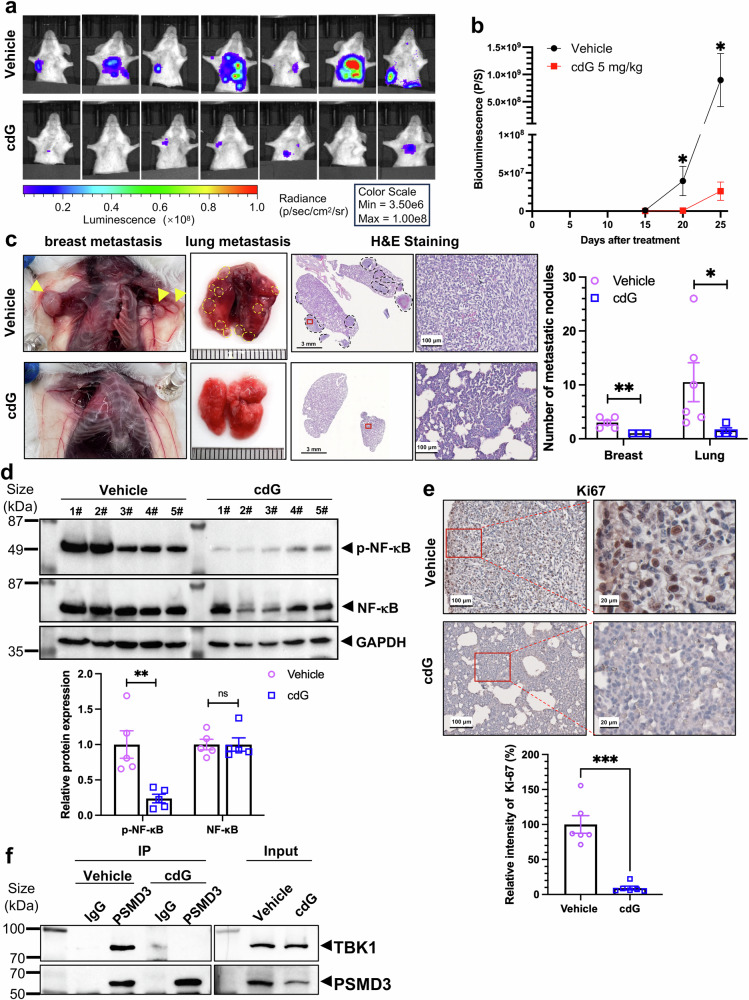
c-di-GMP inhibits lung metastasis of mouse orthotopic mammary tumors in vivo. **a** In vivo bioluminescence imaging was used to track the spread of 4T1-luc cells in an orthotopic breast cancer lung metastasis mouse model following c-di-GMP treatment. Since distant metastatic tumors (in the breast and lungs) can be obscured by the primary orthotopic tumor in the chest mammary gland, the primary orthotopic breast tumors were covered with black paper during imaging to enhance visibility. **b** Quantification of bioluminescence (luciferase flux; p/s = photons/second) for lung and breast metastases. *n* = 7 mice per group. The data are presented as the means ± SEMs. *P < 0.05 (one-way ANOVA followed by Bonferroni’s multiple comparison test). **c** Representative photographs showing the gross appearance of tumor nodules in the breast and lungs (left). H&E staining of lung sections from the indicated groups (middle). Scale bar, 3 mm or 100 μm as indicated. The numbers of metastatic nodules in the breast and lung were quantified separately (right). The data are presented as the means ± SEMs. *P < 0.05, **P < 0.01 compared with the vehicle group. **d** c-di-GMP decreases p-NF-κB protein levels in orthotopic tumors, as determined by WB assays with the indicated antibodies. Each number represents a tumor from an individual mouse. **e** IHC staining was used to detect Ki67-positive cells in lung sections from the indicated groups, which were analyzed via ImageJ/FIJI software. The data are presented as the means ± SEMs. ***P < 0.001 compared with the vehicle group. Scale bar, 100 μm or 20 μm as indicated. **f** c-di-GMP treatment reduces the interaction between endogenous PSMD3 and TBK1 in mouse orthotopic tumor tissues

To mimic human breast cancer metastasis and further validate the antimetastatic effects of c-di-GMP, we employed an orthotopic mouse model of spontaneous breast cancer metastasis. Luciferase-labeled mouse mammary tumor 4T1 cells were injected into the mouse mammary gland under the fourth major nipple.^38^ Two weeks post-implantation, lung metastatic tumors were detectable, and c-di-GMP treatment (5 mg/kg via i.p.) initiated five days post-implantation significantly reduced distant (breast-to-breast and breast-to-lung) metastasis by 34-fold compared with that in the vehicle group. The tumor burden at metastatic sites was evaluated by measuring the bioluminescence intensity via IVIS and quantified (Fig. 7a, b). In the vehicle-treated group, extensive distant metastases were observed in the breast and lungs, with numerous tumor nodules. In contrast, the c-di-GMP treatment group presented barely any detectable tumor nodules in the lungs or other organs (Fig. 7c and Supplementary Fig. 12e). Histological analyses confirmed these findings, as H&E staining revealed a marked reduction in metastatic cancer cells in the lungs of c-di-GMP-treated mice (Fig. 7c). Additionally, IHC staining for Ki-67 revealed a significant reduction in proliferative signals in lung tissues from the c-di-GMP-treated group (Fig. 7e), further confirming the potent antimetastatic effect of c-di-GMP.

While c-di-GMP also inhibited the growth of primary orthotopic tumors, the reduction was less pronounced than its antimetastatic effect (Supplementary Fig. 12a–c). Ki-67 staining of orthotopic tumors did not significantly differ between the c-di-GMP-treated and vehicle-treated groups (Supplementary Fig. 12d). Moreover, c-di-GMP treatment markedly decreased p-NF-κB (p-p65) protein levels in orthotopic tumors (Fig. 7d) and reduced the binding between endogenous PSMD3 and TBK1 in orthotopic tumor tissues (Fig. 7f), resulting in the suppression of primary tumor cell dissemination to distal organs. These results are consistent with the results obtained in cultured cells (Figs. 3d and 5c). In line with the results above (Figs. 1–7), our findings demonstrate that c-di-GMP significantly suppresses breast cancer lung metastasis in vivo, leading to a reduced tumor burden and decreased proliferation at metastatic sites through inhibition of the NF-κB signaling pathway.

## Discussion

Metastasis is an extraordinarily complex and incompletely understood process in tumor progression involving multiple cellular mechanisms, including detachment from the primary tumor, invasion into surrounding tissues, evasion of immune surveillance, and regulation of the tissue microenvironment.^1,2,5^ The diagnosis of metastatic cancer is considered the final stage in most cancer types and remains a leading cause of cancer-related mortality.^1,2^ The incidence of breast cancer has shown a persistent increasing trend, increasing by 1% annually between 2012 and 2021, with particularly steep increases observed in women under 50 years of age, including 1.4% annually among White women and 2.7% among Asian American/Pacific Islander women. The 5-year survival rate for patients with distant-stage breast cancer (metastatic breast cancer) is only 32%.^39^ Patients with metastatic breast cancer are generally considered incurable, highlighting the urgent need for novel therapeutic strategies and clinical trials aimed at improving patient outcomes.

To address this urgent need, we employed preclinical in vitro and in vivo metastatic models to evaluate potential therapeutic candidates and identified a bacterial intracellular signaling molecule, c-di-GMP, as an effective inhibitor of cancer metastasis. First, c-di-GMP drastically inhibited cancer cell migration at low doses without inducing adverse side effects (Figs. 1 and 7). Notably, this inhibitory effect was not limited to breast cancer cells, as c-di-GMP also dramatically suppressed the migration of melanoma, pancreatic and colorectal cancer cells (Supplementary Figs. 2, 4). To our surprise, even though c-di-GMP is a well-studied ligand of STING,^14^ its antimetastatic effect is independent of STING, as c-di-GMP still inhibits cancer cell migration when STING is depleted via CRISPR (Fig. 2d). STING, a widely expressed protein in both immune and nonimmune cells, plays a critical role in modulating innate immune responses. In cancer, STING expression varies across tumor types but is frequently suppressed or lost in advanced disease stages.^23^ Furthermore, other high-affinity STING ligands, including 2′3’-cGAMP and c-di-AMP, failed to exhibit similar antimetastatic effects (Fig. 2e). These results suggest that c-di-GMP exerts its antimetastatic effects through a STING-independent mechanism.

Indeed, our biotin-c-di-GMP pull-down analysis coupled with mass spectrometry identified PSMD3 as a novel target of c-di-GMP (Fig. 4). This finding was surprising, as PSMD3 is a non-ATPase subunit of the 19S regulatory lid within the 26S proteasome^17,18^^,^ and its 26S proteasome-independent biochemical activity has not been intensively explored. Our further studies revealed a previously unrecognized role of PSMD3 in cancer metastasis (Fig. 6), which aligns with the findings of a recent in vitro study suggesting that PSMD3 promotes cell migration via the deubiquitylation and stabilization of ILF3 in cultured lung cancer cells.^22^ Moreover, SPR and ITC assays confirmed the specific binding between c-di-GMP and PSMD3, with a binding affinity of 4–8 × 10^−6 ^M (Fig. 4d, f), a range consistent with the physiological affinity of other known c-di-GMP-binding proteins.^33^ In contrast, no measurable binding response was observed for 2’3’-cGAMP (Fig. 4e, g). These findings suggest that the antimetastatic effect of c-di-GMP is distinct from its previously reported role in STING activation. Interestingly, c-di-GMP inhibited TBK1 phosphorylation via NF-κB signaling, blocked the nuclear translocation of p-NF-κB (p-p65) (Fig. 3d, e), and downregulated the expression of downstream target genes, chemokines and cytokines (Fig. 3f). Our findings align with those of two other studies showing that PSMD3 plays oncogenic roles in chronic myeloid leukemia (CML) by stabilizing NF-κB^19^ and in breast cancer progression by stabilizing HER2 and preventing its degradation.^20^ Because c-di-GMP does not increase the levels of several 26S proteasome-sensitive proteins in cancer cells (Figs. 2, 3, and 6), it is unlikely that c-di-GMP directly inhibits the proteolytic activity of the 26S proteasome. Furthermore, TBK1 was found to specifically associate with PSMD3 but not with other 19S proteasome subunits, such as PSMC4 or PSMD4 (Fig. 5 and supplementary Fig. 9c, d). This selective interaction suggests that proteasome-free PSMD3 may play a noncanonical, proteasome-independent role in modulating TBK1 activity, which is distinct from its role in proteasomal degradation. Our protein‒protein interaction domain-mapping analyses (Fig. 5d–f) further revealed that PSMD3 interacts with TBK1 within its α-helical scaffold dimerization domain (coiled-coil region; Fig. 5d–f), which could mediate the formation of the compact TBK1 dimer essential for activation. These findings suggest that PSMD3 binding promotes the recruitment and close association of TBK1 dimers, increasing the local concentration of TBK1 and enhancing interdimer interactions that facilitate trans-autophosphorylation and activation. Moreover, PSMD3 depletion affected neither the protein levels of c-Myc (Supplementary Fig. 10b), a short-lived oncoprotein typically degraded via ubiquitin-mediated proteolysis,^40^ nor the total protein levels of TBK1 and NF-κB (Fig. 6a), further supporting a proteasome-independent role of PSMD3. Our bioinformatic analysis of clinical data from publicly available databases revealed that PSMD3 is overexpressed in breast cancer tissues and is correlated with poor prognosis (Fig. 6i, j, and Supplementary Fig. 11a–c). This finding is consistent with previous reports showing that PSMD3 is upregulated in breast tumors compared with normal tissues and that high levels of PSMD3 expression are associated with poor recurrence-free survival (RFS) and distant metastasis-free survival (DMFS).^41^ Together with the literature, our results suggest that PSMD3 plays a critical and noncanonical proteasome-independent role in breast cancer progression and metastasis via regulation of the TBK1-NF-κB pathway (Fig. 6h). Moreover, we identified c-di-GMP as a specific PMSD3-targeted antimetastatic agent, highlighting its potential for developing effective therapies against metastatic cancers.

The antimetastatic effect of c-di-GMP has biological relevance in mouse models and potential clinical applications for human breast cancer as well as other aggressive cancers, such as melanoma and pancreatic, liver, and lung cancers. First, using a lung metastasis mouse model (Figs. 1c–f and 7), we demonstrated that c-di-GMP significantly inhibits cancer metastasis to the lungs by suppressing the NF-κB pathway (Fig. 7d). Targeting the NF-κB signaling pathway has been a major focus of cancer drug discovery given its central role in inflammation and tumor progression. However, many NF-κB inhibitors have largely failed in clinical trials because of their toxicity, limited efficacy, and biological complexity. NF-κB is essential for normal immune and tissue homeostasis; thus, systemic inhibition often causes severe immunosuppression and hepatotoxicity.^42,43^ The pathway’s context-dependent roles—pro-tumorigenic in some cells but tumor-suppressive or immune-stimulatory in others—lead to unpredictable outcomes when this pathway is globally inhibited.^44^ Current strategies emphasize selective, cell type specific, or combinatorial modulation of NF-κB, offering the potential for safer and more effective cancer treatments rather than global suppression. Our study revealed that the therapeutic effect of c-di-GMP was evident even at low doses (1 and 5 mg/kg) in animals. According to FDA guidelines (https://www.fda.gov/regulatory-information/search-fda-guidance-documents/estimating-maximum-safe-starting-dose-initial-clinical-trials-therapeutics-adult-healthy-volunteers) for converting animal doses to human equivalent doses on the basis of body surface area, a 5 mg/kg dose of c-di-GMP in mice corresponds to an approximate dose of 25 mg administered every other day for a 60 kg adult. Additionally, no apparent toxicity was observed in mice treated with 10 mg/kg c-di-GMP for six weeks (Supplementary Fig. 3). Furthermore, the observed antimetastatic effect was primarily due to its inhibition of NF-κB-mediated cancer cell migration, as c-di-GMP dramatically reduced the number of distant (breast-to-lung and breast-to-breast) metastatic tumors by more than 34-fold (Fig. 7b). In contrast, it only moderately reduced the size of primary orthotopic breast tumors by ~30% (Supplementary Fig. 12a–c).

Taken together, our findings open a new avenue for investigating the novel antimetastatic function of c-di-GMP and highlight its potential as a broad-spectrum, well-tolerated, and highly effective antimetastatic agent. This warrants further exploration of its mechanisms and in vivo applications in preclinical and clinical settings. Further studies are needed to gain deeper molecular insights into PSMD3-mediated metastasis and its regulation of the TBK1-NF-κB pathway (Fig. 6h) and explore the therapeutic potential of combining c-di-GMP with a PSMD3 inhibitor, a proteasome-targeting agent, chemotherapy or immunotherapy for treating metastatic breast cancer and other metastatic cancers. Finally, whether the PSMD3-TBK1 complex might play a role in NF-κB activation in normal immune cells upon pathological stress and/or in nonimmune cells under physiological conditions is unknown.

## Materials and methods

### Cell lines and culture

Various human cancer cell lines, such as MDA-MB-231 (RRID:CVCL_0062), MDA-MB-231-Luc2 (RRID:CVCL_D582), BT-549 (RRID:CVCL_1092), MCF-7 (RRID:CVCL_0031), Hs578T (RRID:CVCL_0332), PANC-1 (RRID:CVCL_0480), LoVo (RRID:CVCL_0399), H1299 (RRID:CVCL_0060), Calu-1 (RRID:CVCL_0608), B16-F10-Luc (JCRB Cat# NIHS0699, RRID:CVCL_4Y00) and HEK293T (ATCC Cat# CRL-3216, RRID:CVCL_0063), were cultured in Dulbecco’s modified Eagle’s medium (DMEM) supplemented with 10% fetal bovine serum (GIBCO), 1% penicillin and streptomycin (GIBCO). AsPC-1 (RRID:CVCL_0152) and 4T1-Luc (RRID:CVCL_J239) cells were maintained in RPMI-1640 medium with the same supplements. All the cells were grown at 37 °C in a humidified incubator with a 95:5 (%; v/v) air-to-CO_2_ ratio. STR profiling was performed to ensure cell identity, and no mycoplasma contamination was detected.

### Wound healing and transwell assays

Tumor cell migration was assessed via wound healing and transwell assays.^45^ For the wound-healing assay, confluent cells in 6-well plates were scratched with a pipette tip, treated with medium containing 10% FBS and c-di-GMP, and incubated for 16–24 h. The cells were then fixed with 3.7% paraformaldehyde, photographed, and manually quantified. Migration inhibition was calculated as 100% in the untreated group. The IncuCyte® WoundMaker tool was also used for automated wound healing assays. The cells were seeded into 96-well plates at 30,000 cells per well. Upon reaching 100% confluency, scratch wounds were generated via the IncuCyte Wound Maker Kit, followed by continued drug treatment for 20 h. Images were captured via the IncuCyte Live-Cell Analysis System, and the wound width was analyzed via built-in software (RRID:SCR_026298).

For the transwell migration assay, 5–10 × 10^4^ cells per well were pretreated with the indicated treatment and seeded into the upper chamber of 24-well cell culture inserts^45^ (8-μm pore; FALCON) in 100 μL of serum-free medium. The lower chamber was filled with 500 μL of medium containing 10% FBS as a chemoattractant. After incubation, the migrated cells on the underside of the membrane were fixed, stained with 1% crystal violet, counted in six random fields per insert via ImageJ software and normalized to the control. At least two inserts for each condition were analyzed.

### Cell viability assay

The cells were plated at a density of 2000–5000 cells per well in 96-well plates and allowed to adhere overnight. The indicated treatments were then applied, and cell viability was determined via the Cell Counting Kit-8 (CCK-8) (Dojindo) according to the manufacturer’s instructions or via the Sartorius IncuCyte SX5 Live Cell Analysis System (RRID:SCR_026298).^46^ The half-maximal inhibitory concentration (IC_50_) values were calculated via Prism 10 (GraphPad Prism, RRID:SCR_002798).

### Mouse xenograft experiments

Cancer lung metastasis mouse models were established as described previously.^45^ All animal experiments were approved by East China Normal University (Shanghai, China) and conducted in accordance with the Institutional Animal Care and Use Committee guidelines. BALB/cA nude mice (RRID:IMSR_CRL:194) and C57/BL6 mice (RRID:IMSR_JAX:000664) were purchased from National Rodent Laboratory Animal Resources (Shanghai, China) and maintained in a laminar airflow cabinet under specific-pathogen-free conditions. For the lung metastasis model, MDA-MB-231-Luc cells (1 × 10^6^ cells in 100 μL of PBS) were injected into the lateral tail vein of female nude mice. B16-F10-luc murine melanoma cells (1 × 10^5^ cells in 100 μL of PBS) were injected into the lateral tail vein of female C57BL/6 mice. The mice were divided into two or three groups (*n* = 8 per group) on the basis of the initial IVIS image: (1) control group: PBS, every other day (i.p.); (2) low-dose c-di-GMP group: 1 mg/kg every other day (i.p.); and (3) high-dose c-di-GMP group: 10 mg/kg every other day (i.p.). Tumor metastasis to the lungs was determined weekly via bioluminescence imaging via a Xenogen IVIS-200 optical in vivo imaging system (PerkinElmer).

Orthotopic breast cancer lung metastasis models were established as described previously.^38^ Briefly, 4T1-luc mouse mammary tumor cells (1 × 10^5^ in 100 μL of PBS) were implanted subcutaneously under the fourth mammary nipple of 6-week-old female BALB/c mice (RRID:IMSR_JAX:000651). Five days later, the mice were divided into two groups (*n* = 7 per group) on the basis of initial bioluminescence and treated with vehicle or c-di-GMP (5 mg/kg, i.p.) every other day for ~1 month. The orthotopic tumor volume was measured every other day via calipers and calculated as follows: Volume = Length × Width^2^ × 0.52. Lung and distal breast metastases were monitored every 5 days via IVIS imaging, with orthotopic tumors masked to isolate lung signals. All animal experiments were conducted under protocol #1477 approved by the Tulane IACUC.

### Hematoxylin and eosin (H&E) staining and immunohistochemistry (IHC)

Hematoxylin and eosin (H&E) staining and immunohistochemistry (IHC) staining were performed as described previously.^46^ Briefly, the tissues were fixed in formalin, embedded in paraffin, and sectioned at a thickness of 5 μm. The sections were stained with hematoxylin and eosin via a commercially available protocol. For IHC staining, antigen retrieval was performed by steaming the slides in 10 mM sodium citrate. After cooling, endogenous peroxides were inactivated by incubating the slides in 3% hydrogen peroxide for 10 min. The slides were then blocked by incubation with blocking buffer [goat serum in PBST (0.1% Triton X-100 in PBS)] for one hour at room temperature in a humidified chamber. Primary antibodies diluted in blocking buffer were added to the slides, which were subsequently incubated overnight at 4 °C. The slides were then incubated with biotinylated rabbit or mouse (Vector Laboratories) secondary antibodies, which were also diluted in blocking buffer, for one hour at room temperature. After the samples were washed with PBS, developing reagents from ABC kits (Vector Laboratories) were applied. DAB kits (Vector Laboratories) were used for color development. Finally, the slides were costained with hematoxylin, dehydrated, and mounted. The slides were examined under a microscope (IX-71, OLYMPUS) or scanned via the Akoya PhenoImager™ Fusion platform and analyzed via ImageJ/FIJI software as described previously.^47^

### RNA-sequencing and quantitative PCR analysis

The RNA-sequencing service was provided by Novogene. Total RNA was extracted from MDA-MB-231 cells treated with 5 μM c-di-GMP for 24 h and from the vehicle control group (*n* = 4). Differentially expressed genes (DEGs) were identified via DESeq2 (1.20.0). DEG, KEGG and GSEA analyses were carried out via the NovoMagic platform (Novogene Co.). GSEA generates a normalized enrichment score (NES) to evaluate the extent to which a specific pathway is enriched in a ranked gene list compared with a randomly distributed background. An NES > 0 indicates pathway activation, whereas an NES < 0 reflects pathway repression relative to the reference group. Both NES and p values were used to assess the statistical significance of pathway enrichment.

Reverse transcription and RT–qPCR for mRNAs were performed via methods described previously.^48^ Briefly, total RNA was extracted from cells via TRIzol (Invitrogen) according to the manufacturer’s instructions. 0.5–1 μg of RNA was reverse-transcribed into cDNA using poly-(T)20 primers and M-MLV reverse transcriptase (Promega). Quantitative PCR (qPCR) was then performed via SYBR Green Mix (Bio-Rad) following the manufacturer’s guidelines. All reactions were carried out in triplicate, and the sequences of primers used are listed in Supplementary Table 3.

### Plasmids

The full-length HA-TBK1 construct was generated via the pHAGE-N-FLAG-HA-TBK1 plasmid (RRID: Addgene_131791) as the cDNA template and cloned and inserted into the pcDNA3.1-HA (RRID: Addgene_128034) vector for cellular overexpression. The His-tagged full-length and truncated TBK1 constructs were generated in our laboratory by cloning into the pET-28a (+) vector for in vitro bacterial expression. His-tagged TBK1 protein was expressed in *E. coli* BL21 cells induced with 1 mM isopropyl-β-D-thiogalactoside (IPTG) at 16 °C overnight and purified via HisPur™ Ni-NTA Resin (Thermo Scientific, 88221).

The human PSMD3 ORF vector was purchased from Applied Biological Materials Inc. (ABM, #38002013). The full-length PSMD3 and its fragments were constructed in our laboratory and cloned and inserted into a 2xFLAG-pcDNA 3 vector backbone for mammalian expression and a pET-28a (+) vector backbone (EMD Biosciences, Cat# 69864-3) for in vitro bacterial expression.

### Western blotting

Western blotting (WB) assays were carried out according to established procedures with minor modifications.^49^ Following the indicated treatments or plasmids/siRNA transfections, whole-cell lysates or tumor tissue samples were prepared with radioimmunoprecipitation (RIPA) buffer (150 mM NaCl, 50 mM Tris, 1% Triton X-100, 0.5% deoxycholate, and 0.1% SDS) supplemented with protease inhibitors, and incubated on ice for 30 min. Equal amounts of lysate (30–50 μg) were separated by 8–12% SDS‒PAGE, followed by transfer onto polyvinylidene difluoride (PVDF) membranes (Bio-Rad, 1620177). Membranes were blocked with 5% non-fat milk for 1 h at room temperature, and then incubated with the indicated primary antibodies overnight at 4 °C, washed, and incubated with secondary antibodies. Signal was detected using ECL substrate (Thermo Scientific, 34578) and imaged with the Bio-Rad ChemiDoc MP Imaging System (RRID:SCR_019037). Representative blots from several independent experiments are shown, and band intensities were quantified via ImageJ and normalized to that of the control. A complete list of antibodies with RRIDs is provided in Supplementary Table 1.

### Immunoprecipitation

For standard immunoprecipitation (IP) assays followed by WB (IP-WB), cells or tumor tissues subjected to the indicated treatments or transfections were lysed in lysis buffer [50 mM Tris-HCl pH 7.5, 150 mM NaCl, 5 mM EDTA, 0.5% NP40, 1 mM DTT, 0.2 mM phenylmethylsulfonyl fluoride (PMSF) and protease inhibitors]. The lysates were cleared by centrifugation at 12,000 rpm for 20 min at 4 °C to remove cell debris. For immunoprecipitation, 500–1000 μg of total protein was incubated with a specific antibody or control IgG (Thermo Fisher Scientific Cat# 31903, RRID:AB_10959891) overnight at 4 °C. The immune complexes were then incubated with protein G PLUS-Agarose (Santa Cruz Biotechnology Cat# sc-2002, RRID:AB_10200697) or protein A-Agarose (Santa Cruz Biotechnology Cat# sc-2001, RRID:AB_10201241) for an additional 1–2 h. The Flag-tagged and HA-tagged proteins were immunoprecipitated via Flag beads (Sigma-Aldrich Cat# A2220, RRID:AB_10063035) or HA beads (GenScript Cat# L00777, RRID:AB_3677368), respectively. After three washes in lysis buffer, the immunoprecipitates were analyzed via WB.

### Pull-down and MS analysis of c-di-GMP-bound proteins

To identify potential c-di-GMP target proteins,^50^ MDA-MB-231 cell lysates were incubated overnight at 4 °C with biotin or biotinylated c-di-GMP (8-biotin-11-c-diGMP; BIOLOG Cat. No. B 184) in the absence or presence of a 20-fold excess of unlabeled c-di-GMP. The protein complexes were then captured using NeutrAvidin™ Agarose (Thermo, 29200) for 4 h at 4 °C. Bead-bound proteins were separated via SDS‒PAGE and visualized via silver staining. The specific protein-containing band in the biotin-c-di-GMP lane was excised and subjected to in-gel digestion and LC‒MS/MS analysis, which was conducted at the OHSU Proteomics Shared Resource Core Facility (RRID:SCR_009991).

### Cellular thermal shift assay (CETSA)

The cell lysate CETSA experiments were performed as previously described.^51^ Briefly, MDA-MB-231 cells were harvested and washed with PBS supplemented with complete protease inhibitor cocktail. The cell suspensions were subjected to three freeze‒thaw cycles in liquid nitrogen. The soluble fraction (lysate) was then separated from the cell debris by centrifugation at 20,000 × *g* for 20 min at 4 °C. The cell lysates were divided into two aliquots, with one aliquot being treated with c-di-GMP and the other with the diluent of the chemical (PBS control). After 30 min of incubation at room temperature, the lysates were further divided into smaller (50 μL) aliquots and heated individually at different temperatures for 3 min via a Bio-Rad thermal cycler, followed by cooling for 3 min at room temperature. The heated lysates were then centrifuged at 20,000 × *g* for 20 min at 4 °C to separate the soluble fractions from the precipitated proteins. The supernatants were transferred to new microtubes and analyzed by Western blotting.

### Surface plasmon resonance (SPR) binding assay

SPR experiments were performed on a Biacore T200 SPR system (Cytiva; RRID:SCR_019718) at 25 °C, with data collected at 1 Hz. Recombinant PSMD3 E61–S460 was immobilized on a CM5 sensor chip (Cytiva; RRID:SCR_023581) via amine coupling using the manufacturer’s EDC/NHS chemistry to a level of ~5000 RU. A reference channel was prepared by activating and deactivating the chip surface without protein. Analytes (c-di-GMP or 2’3’-cGAMP) were prepared as stock solutions and diluted in running buffer (10 mM HEPES, pH 7.4, 150 mM NaCl, 0.005% Tween-20) to yield a six-point, twofold serial dilution (125 μM–3.9 μM) for c-di-GMP and a seven-point, twofold serial dilution (250 μM–3.9 μM) for 2’3’-cGAMP.

Binding affinity measurements were collected in three independent repeats in a multicycle kinetics experiment. Each analyte was injected sequentially at 30 μL·min^−1^ from low to high concentrations in a multicycle kinetic format with a 60 s association and 360 s dissociation phase. Three buffer-only injections were included as blanks at the start and end of the run.

Sensorgrams were double-referenced against the control channel and buffer cycle via BIAEvaluation software (version 3.1). Global fitting to a 1:1 binding model yielded kinetic parameters and an apparent dissociation constant (K_*d*_). Representative sensorgrams were plotted in GraphPad Prism (version 10.4.0).

### Isothermal titration calorimetry

Isothermal titration calorimetry (ITC) experiments were performed on a *MicroCal iTC200* microcalorimeter (Malvern Panalytical) at 25 °C. Recombinant PSMD3 E61–S460 was dialyzed extensively against ITC buffer (20 mM HEPES, pH 7.4; 150 mM NaCl; 2 mM MgCl_2_). Protein and ligand solutions were prepared in the same freshly degassed buffer immediately before use. The syringe contained c-di-GMP (500 μM) or 2′,3′-cGAMP (1 mM), and the sample cell contained protein (65 μM).

The titrations consisted of an initial 0.5–1 μL injection followed by 18 injections of 2 μL each at 220–300 s intervals, with stirring at 500 rpm. Baseline equilibration was confirmed prior to each experiment. Integrated heats were analyzed in Origin 7.0 (OriginLab) and fitted to a single-site binding model to determine thermodynamic parameters (K_*d*_, ΔH, ΔS, and *n*). Control titrations of the ligand into the buffer were subtracted to correct for heats of dilution. Binding affinity measurements were performed in three independent repeats.

The GRP78 protein served as a nucleotide-binding negative control. GRP78 was expressed and purified as previously described,^52,53^ dialyzed into the same ITC buffer, and analyzed under identical conditions.

### RNA interference and CRISPR/Cas9-mediated gene editing

For RNA interference experiments, cells were transfected with 50–100 nM small interfering RNA (siRNA) or a corresponding negative control (NC) siRNA via TurboFect transfection reagent (Thermo Scientific), according to the manufacturer’s instructions. Following 48–72 h of transfection, the cells were harvested for subsequent analyses, including cell viability assays, western blotting to assess the knockdown efficiency or processed for RT‒qPCR. All siRNAs were synthesized and purified by Dharmacon.

To generate the STING knockout cell lines, CRISPR/Cas9-mediated genome editing was employed. Single-guide RNAs (sgRNAs) targeting STING were designed via the following methods: http://crispr.mit.edu/. The lentiCRISPR v2 plasmid (RRID: Addgene_52961) containing *STING* sgRNA or control sgRNA (sg-Ctrl) was packaged within the lentivirus and used to infect MDA-MB-231 cells. Following infection, single-cell colonies were isolated, expanded, and validated for STING knockout (KO) by WB analysis. The sequences of all siRNAs and sgRNAs used in this study are listed in Supplementary Table 2.

### Statistical analysis

The data are presented as the means ± SDs unless otherwise stated. Statistical tests were performed via Microsoft Excel (RRID:SCR_016137) and GraphPad Prism Software (RRID:SCR_002798). For comparisons between two groups, a two-tailed unpaired *t* test was used. For comparisons among multiple groups, one-way analysis of variance (ANOVA) was performed. A log-rank test was performed for survival curve analysis. Statistical significance was defined as *P* < 0.05 (*), *P* < 0.01 (**) and *P* < 0.001 (***). The specific tests used are detailed in the figure legends.

## Supplementary information

Wang et al suppl inform

Wang et al--Suppl raw WB blot data

Supplementary table S1

Supplementary table S2

Supplementary table S3

Supplementary table S4

Supplementary table S5

## Data Availability

All data supporting the findings of this study within the main text and supplementary information are available. The original RNA-sequencing data presented in the study are publicly available. These data can be found on the following webpage, https://www.ncbi.nlm.nih.gov, with the accession number GSE309627. The UALCAN platform (https://ualcan.path.uab.edu; RRID:SCR_015827) and the GEPIA2 database (http://gepia2.cancer-pku.cn/#index; Gene Expression Profiling Interactive Analysis 2 (RRID:SCR_026154)) were used to compare PSMD3 expression levels between tumor and normal tissues in breast invasive carcinoma (BRCA) from the TCGA dataset. Additionally, survival analyses were performed via Kaplan‒Meier Plotter (http://www.kmplot.com/analysis/; RRID:SCR_018753).
