# Supplementary material for: Cyclic di-GMP suppresses cancer metastasis by targeting proteasome 26S subunit non-ATPase 3 independently of STING
Source: Signal Transduct Target Ther. 2026 Feb 4;11:44. doi: 10.1038/s41392-025-02553-9 (PMC12868630; doi:10.1038/s41392-025-02553-9)
Supplement: Supplementary file 1 — Wang et al suppl inform [file 41392_2025_2553_MOESM1_ESM.docx]

Supplementary Materials for

Cyclic di-GMP suppresses cancer metastasis by targeting PSMD3 independently of STING

Jieqiong Wang^1,2^, Alexander Mrozek^1,2^, Kewen Hu^3,4^, Hanyu You^1,2^, Sarah E Traverse^1,2^, Hyemin Lee^1,2^, Shelya X. Zeng^1,2^, Xiufeng Pang^3^, Heewon Park^1,2^, Hua Lu^1,2,*^

Correspondence to: hlu2@tulane.edu

**This Word Doc file includes:**

Supplementary Fig. 1-12

**Other Supplementary Materials for this manuscript include the following:**

Supplementary Tables S1-S5:

Supplementary tables S1_antibodies

Supplementary tables S2_si&sg RNA sequences

Supplementary tables S3_primers

Supplementary tables S4_protein list

Supplementary tables S5_RNA_Seq_DEGs

Supplementary materials_raw data of Western blots

**Supplementary figures**

**
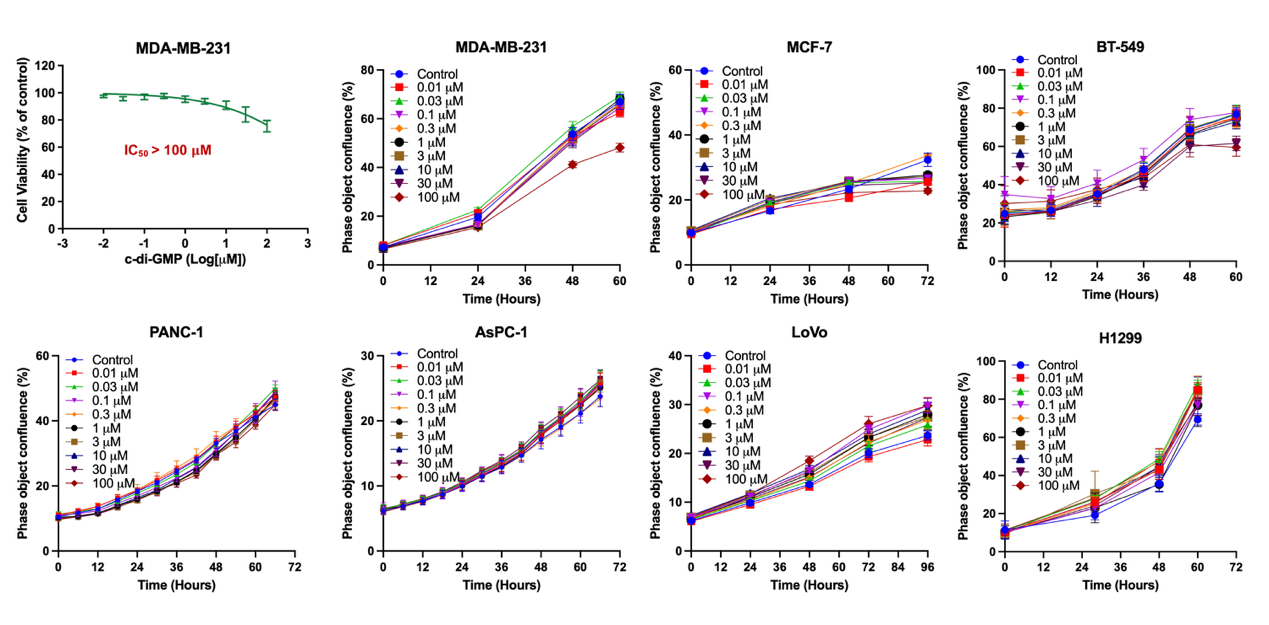
**

**Supplementary Fig. 1, related to Fig. 1: Little and moderate effects of c-di-GMP on cell viability of various cancer cells.** Cancer cells were treated with different concentrations of c-di-GMP for the indicated time. The first figure, MDA-MB-231 cell viability was assessed using the CCK-8 reagent after c-di-GMP treatment for 72 h. All data normalized to vehicle (PBS) control. Dose–response curves and IC_50_ value was generated using GraphPad Prism 5.0. The other figures, the confluence of cancer cell lines was monitored using the IncuCyte over time. Data are presented as mean ± SD.

**
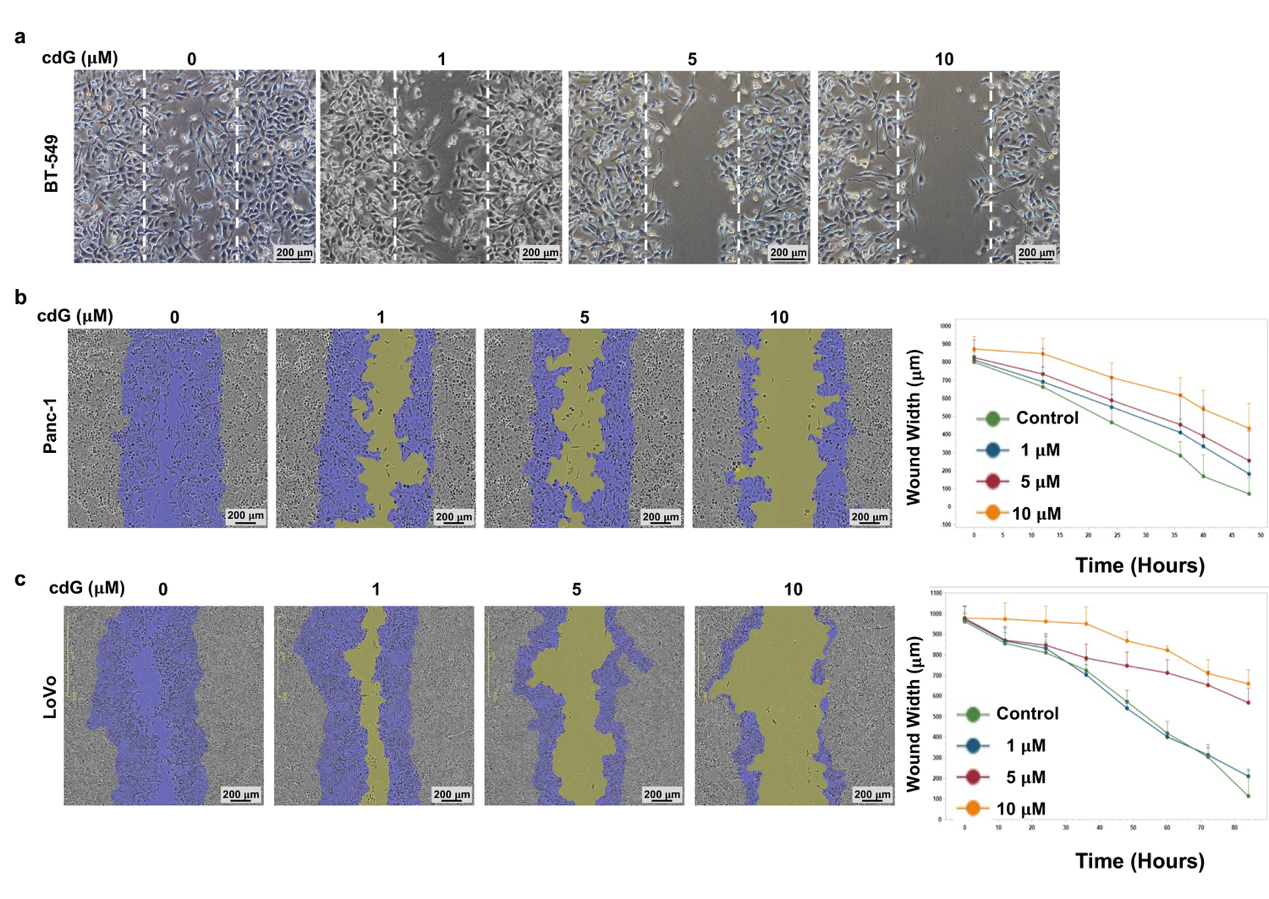
**

**Supplementary Fig. 2, related to Fig. 1: c-di-GMP inhibits cancer cell metastasis in various cell lines. a,** Representative images of a wound-healing assay in BT-549 cells treated with the indicated doses of c-di-GMP for 18 h. Scale bar, 200 μm. **b and c,** Migration of PANC-1 and LoVo cells treated with c-di-GMP at the indicated doses was assessed using a wound healing scratch assay. The IncuCyte WoundMaker kit was used to create wounds, and images were captured using the IncuCyte Zoom system. Scale bar, 200 μm. Wound width was analyzed by the IncuCyte software.


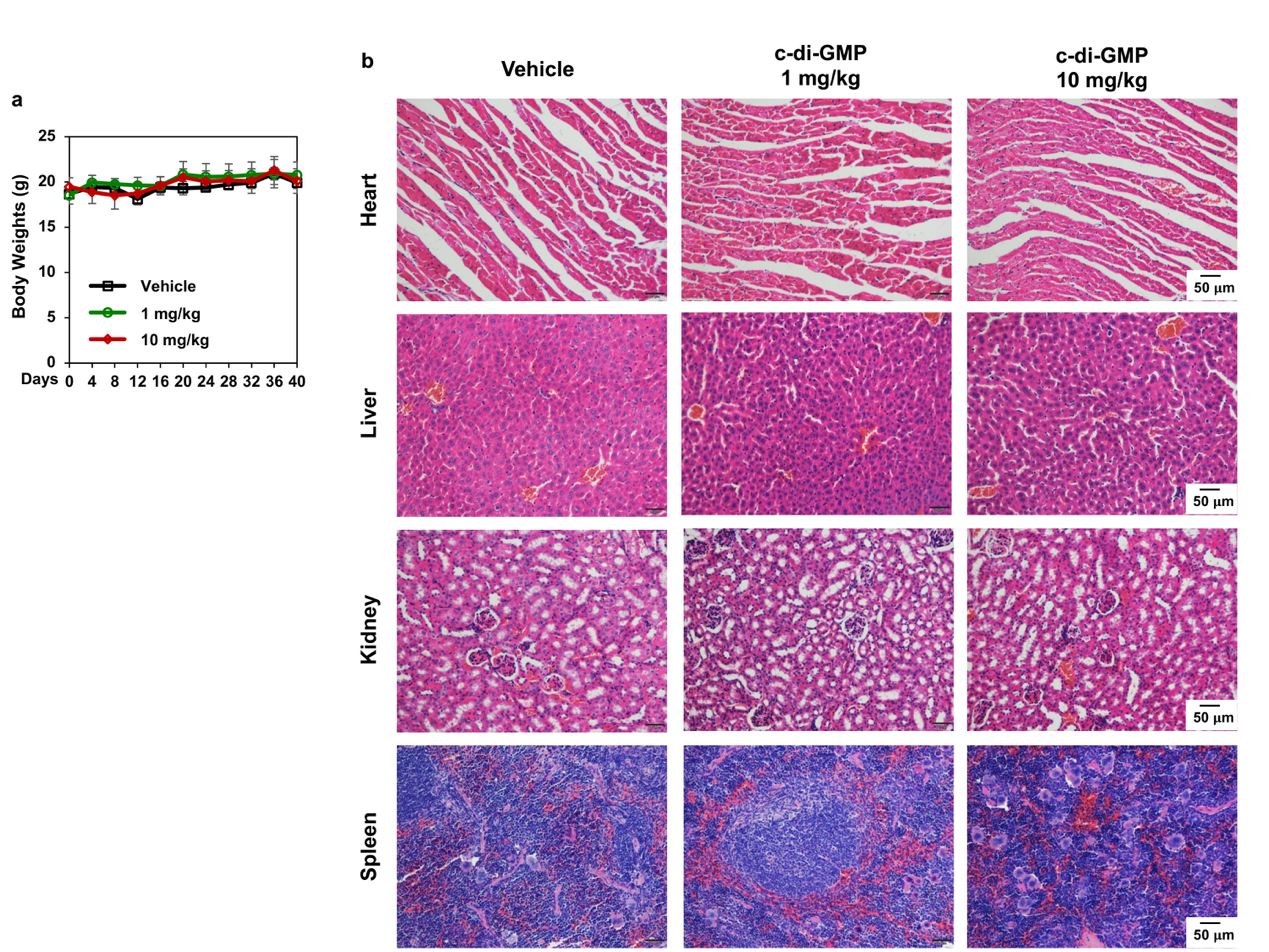


**Supplementary Fig. 3, related to Fig. 1: c-di-GMP suppresses cancer metastasis in a breast cancer lung metastasis model. a,** Mouse body weight in a lung metastasis mouse model after tail vein injection of highly metastatic, luciferase-labeled MDA-MB-231 human breast cancer cells, followed by c-di-GMP treatment for 6 weeks. **b,** H&E staining of major organs (heart, liver, kidney and spleen) from MDA-MB-231 xenograft-bearing mice treated with c-di-GMP (1 mg/kg and 10 mg/kg) or vehicle control. No toxicopathological changes were observed. Scale bar, 50 μm.


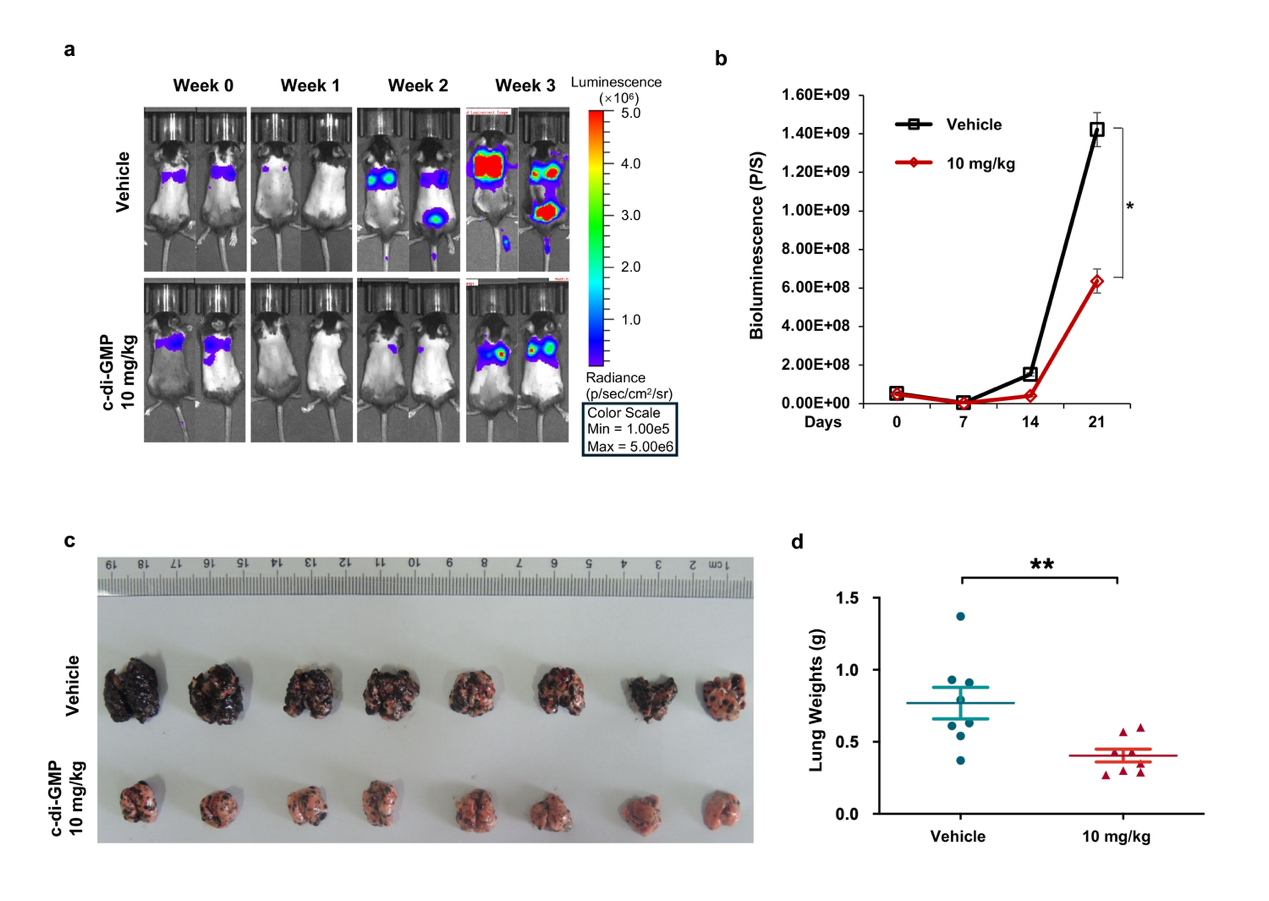


**Supplementary Fig. 4, related to Fig. 1: c-di-GMP suppresses cancer metastasis in a melanoma lung metastasis model. a,** Representative bioluminescent images of metastatic melanoma lesions. Highly metastatic murine melanoma B16-F10 cells were injected intravenously into female C57/BL6 mice. PBS or c-di-GMP (10 mg/kg) was administered intraperitoneally on day 0 and then every other day. Lung metastases were monitored weekly using an in vivo imaging system for 3 weeks. **b,** Quantification of bioluminescence signal (luciferase flux; p/s = photons/second) for lung metastases. n  =  8 mice per group. **c,** Gross anatomy of dissected lung tissues at the endpoint. **d,** Lung weight at the end point of the indicated treatments. Each dot represents an individual mouse. Data are presented as the mean ± SEM. **P < 0.01 by one-way ANOVA followed by Bonferroni’s multiple comparison test.


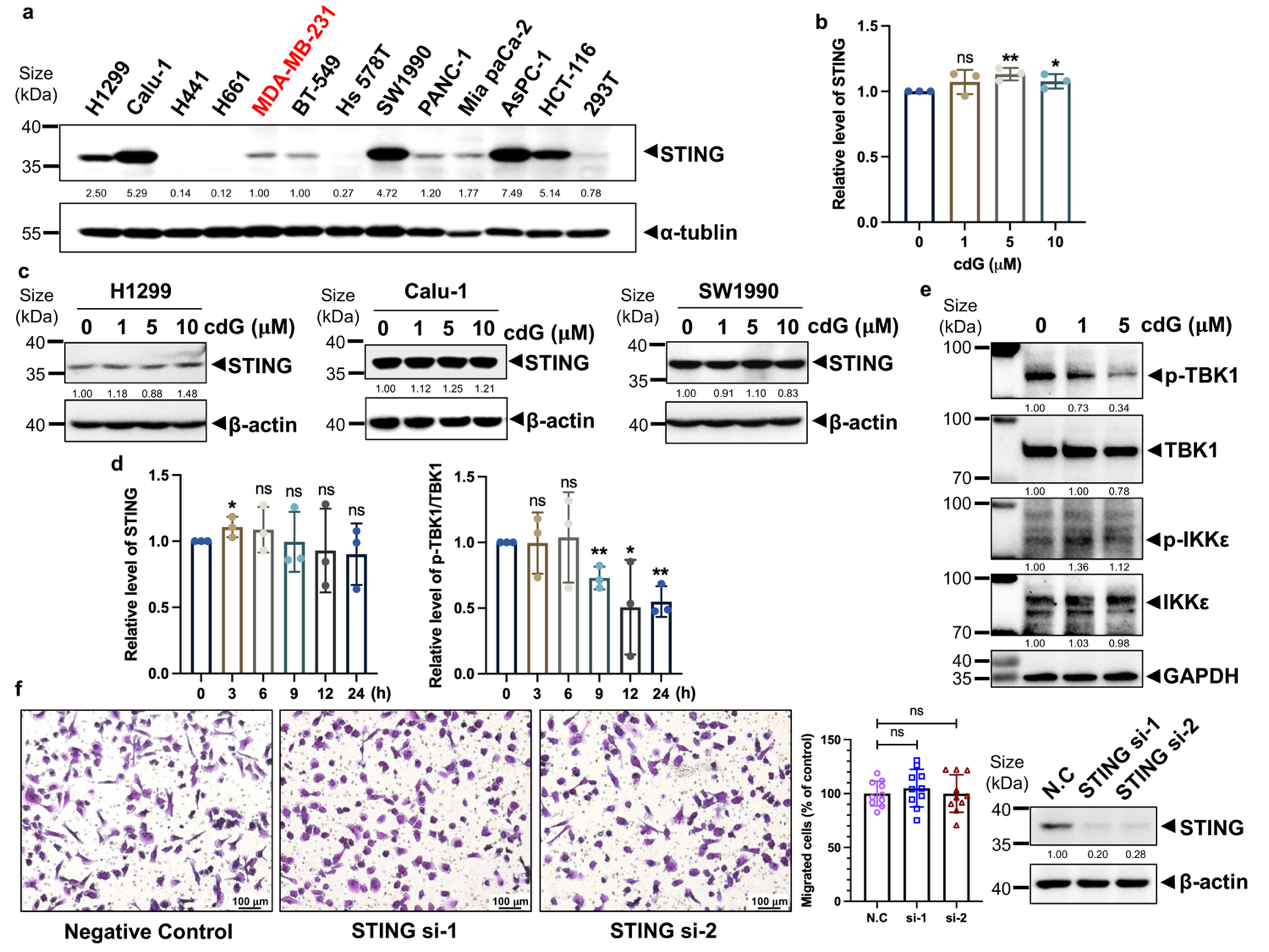


**Supplementary Fig. 5, related to Fig. 2: The anti-metastatic effect of c-di-GMP is independent of STING. a,** Western blots analysis of STING protein expression in various cancer cell lines. HEK293T cells, which lack STING expression, were used as a negative control. **b,** Related to Fig. 2a. Densitometric analysis of bands from three independent experiments was performed using ImageJ. Values were normalized against the corresponding internal control and presented relative to the non-treated group, and shown as mean ± SD. ns, not significant, *p < 0.05, **p < 0.01 by two tailed t-test. **c,** H1299, Calu-1 and SW1990 cells were treated with the indicated doses of c-di-GMP for 24 hours, and total STING levels were assessed by WB analysis. **d,** Related to Fig. 2b. Densitometric analysis of bands from three independent experiments was performed using ImageJ. Values were normalized to the corresponding internal control and presented relative to T0 group, and shown as mean ± SD. ns, not significant, *p < 0.05, **p < 0.01 by two tailed t-test. **e,** MDA-MB-231 cells were treated with increasing concentrations of c-di-GMP for 24 hours, and whole-cell lysates were analyzed by Western blotting. **f,** STING knockdown in MDA-MB-231 cells was achieved by siRNAs. Metastasis was analyzed using a Transwell migration assay. Representative images are shown (left), and the efficiency of STING knockdown was confirmed by western blots (right). Scale bar, 100 μm.


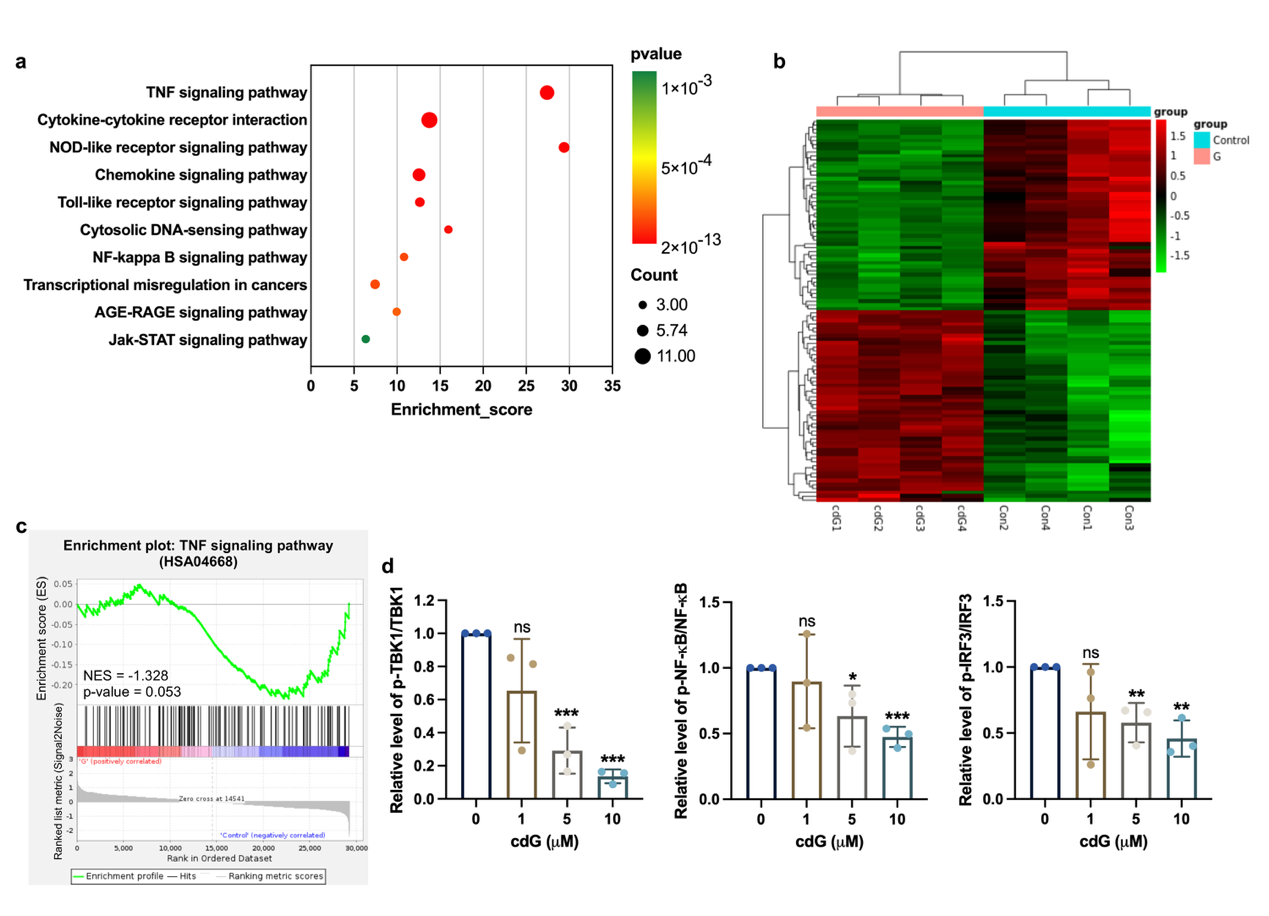


**Supplementary Fig. 6, related to Fig. 3: c-di-GMP inhibits NF-κB signaling pathway. a,** KEGG enrichment analysis of downregulated genes in MDA-MB-231 cells following c-di-GMP treatment (5 μM for 24 hours), as identified by RNA-seq (n = 3; Supplementary Table 5). The top 10 signal pathway are shown. **b,** Total RNAs of c-di-GMP treated (5 μM for 24 hours) and untreated MDA-MB-231 cells were extracted and transcriptomes profiled by RNA-Seq (n = 4, GSE309627). Differentially expressed genes (DEGs) were identified using the DESeq2 R package (1.20.0). Genes with absolute log2 fold-change > 0 and p value < 0.05 were assigned as differentially expressed. The 50 most significantly upregulated and downregulated DEGs after c-di-GMP treatment are shown are presented in a heat map (n = 4). **c,** Gene set enrichment analysis (GSEA) of TNFα signaling pathway between c-di-GMP treatment and control groups (NES = -1.328; p value = 0.053). **d,** Related to Fig. 3d. Densitometric analysis of bands from three independent experiments was performed using ImageJ. Values were normalized against the corresponding internal control and presented relative to the non-treated group, and shown as mean ± SD. ns, not significant, *p < 0.05, **p < 0.01, ***p < 0.001 by two tailed t-test.


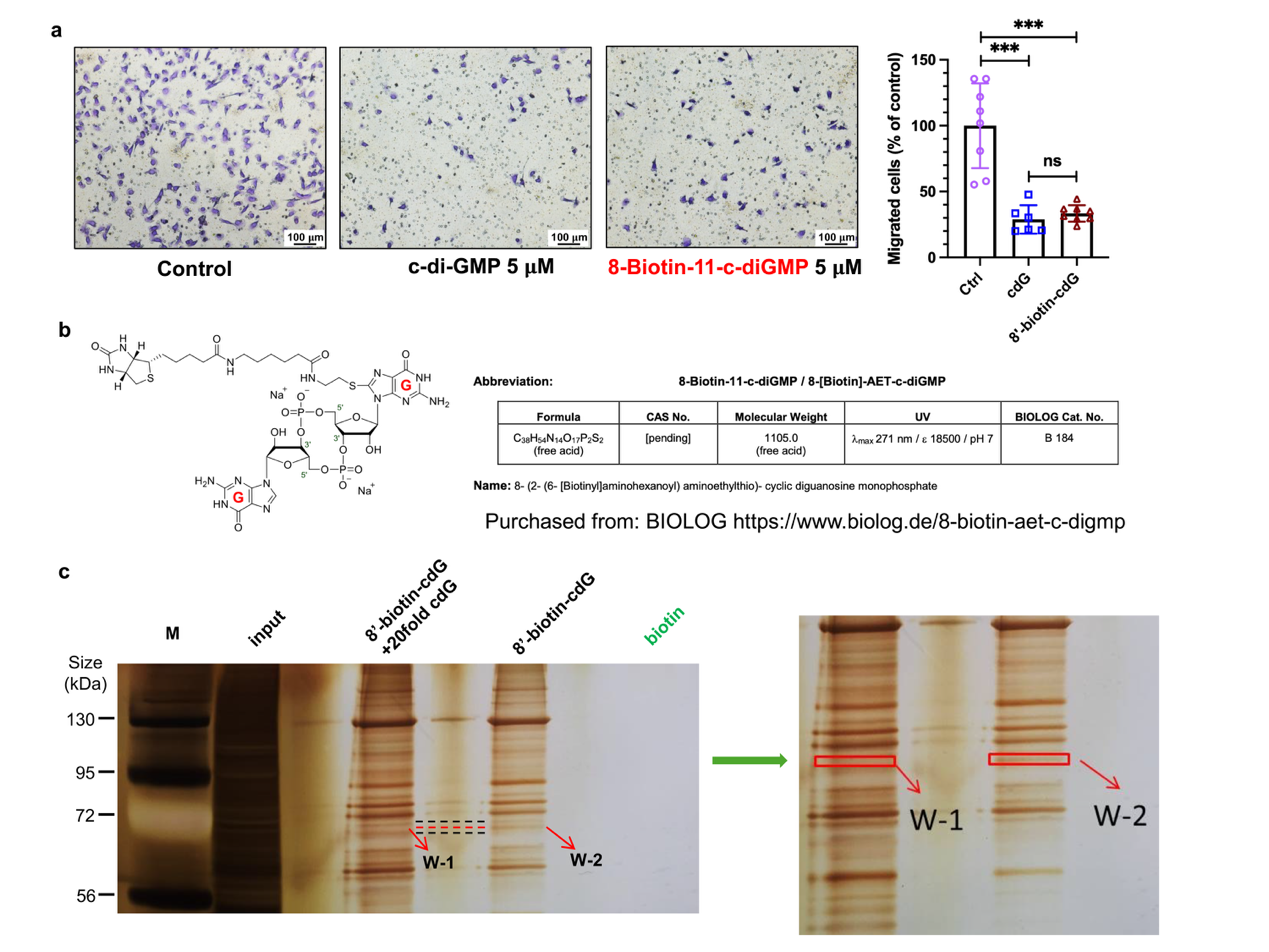


**Supplementary Fig. 7, related to Fig. 4: Biotinylated c-di-GMP exhibits similar metastasis inhibitory effect as c-di-GMP. a,** Migration of MDA-MB-231 cells treated with 5 μM c-d-GMP or biotinylated c-di-GMP (8-Biotin-11-c-di-GMP) for 15-17 h was evaluated using a Transwell assay (left). Scale bar, 100 μm. Migrated cells were quantified manually (right). ***P < 0.001 compared to the control group (right). **b,** Chemical structure of 8-Biotin-11-c-di-GMP, purchased from BIOLOG. <https://www.biolog.de/8-biotin-aet-c-digmp>. **c,** Enlarged and cropped image of Fig. 4a to highlight the specific band precipitated by biotin-c-di-GMP (W-2) (left; pointed by the red dash line). The gel pieces corresponding to the regions indicated by the red frames (W-1 and W-2; right) were excised and then subjected to mass spectrometric analysis to identify c-di-GMP-binding protein.


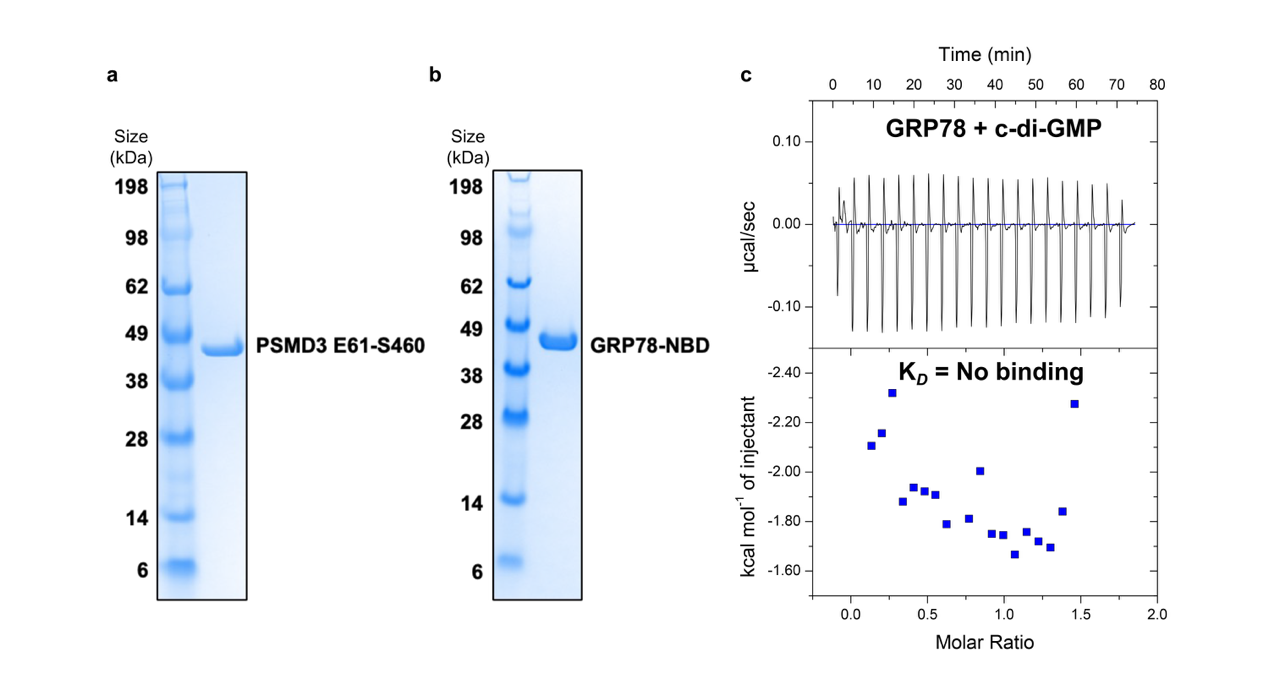


**Supplementary Fig. 8, related to Fig. 4: c-di-GMP does not bind to GRP78 in vitro. a,** **b,** Coomassie Blue–stained SDS-PAGE gels showing purified recombinant human PSMD3 E61–S460 (**a**) and GRP78 nucleotide-binding domain (**b,** GRP78-NBD). Both proteins appear as single bands at their expected molecular weights (~47 kDa and ~44 kDa, respectively), confirming high purity for biophysical analysis. **c,** Isothermal titration calorimetry (ITC) of c-di-GMP titrated into GRP78-NBD under identical conditions used for PSMD3. The raw thermogram (upper) and integrated heats (lower) show no measurable heat exchange or binding isotherm, confirming that GRP78 does not interact with c-di-GMP and validating the specificity of PSMD3–c-di-GMP recognition.


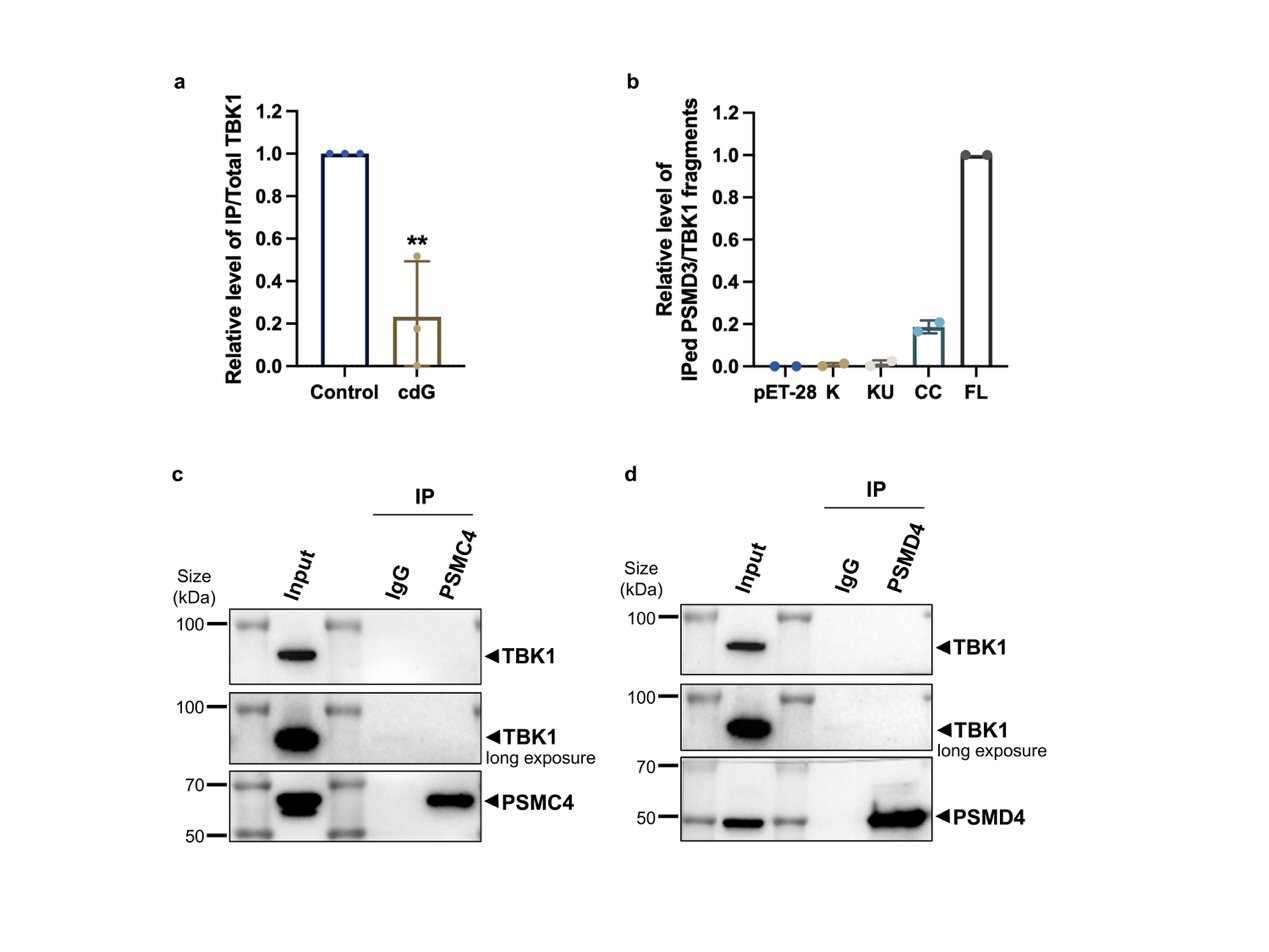


**Supplementary Fig. 9, related to Fig. 5: Other 19S subunits PSMC4 and PSMD4 do not interact with TBK1. a,** Related to Fig. 5c. Densitometric analysis of bands from three independent IP experiments was performed using ImageJ. Relative values were presented as immunoprecipitated (IPed) TBK1 protein/total TBK1 protein, normalized against the non-treated group, and shown as mean ± SD. **p < 0.01 by two tailed t-test. **b,** Related to Fig. 5d. Densitometric analysis of bands from two independent mapping experiments was performed using ImageJ. Relative values with the full-length His-TBK1 group as 1 were presented for IPed flag-PSMD3 with the corresponding His-TBK1 fragment. **c,** 19S proteasome subunit PSMC4 does not interact with TBK1 in MDA-MB-231 cells. PSMC4 antibody and Protein A-Agarose were used for co-IP followed by WB analysis with indicated antibodies. **d,** 19S proteasome subunit PSMD4 does not interact with TBK1 in MDA-MB-231 cells. PSMD4 antibody and Protein A-Agarose were used for co-IP assay followed by WB analysis with indicated antibodies.


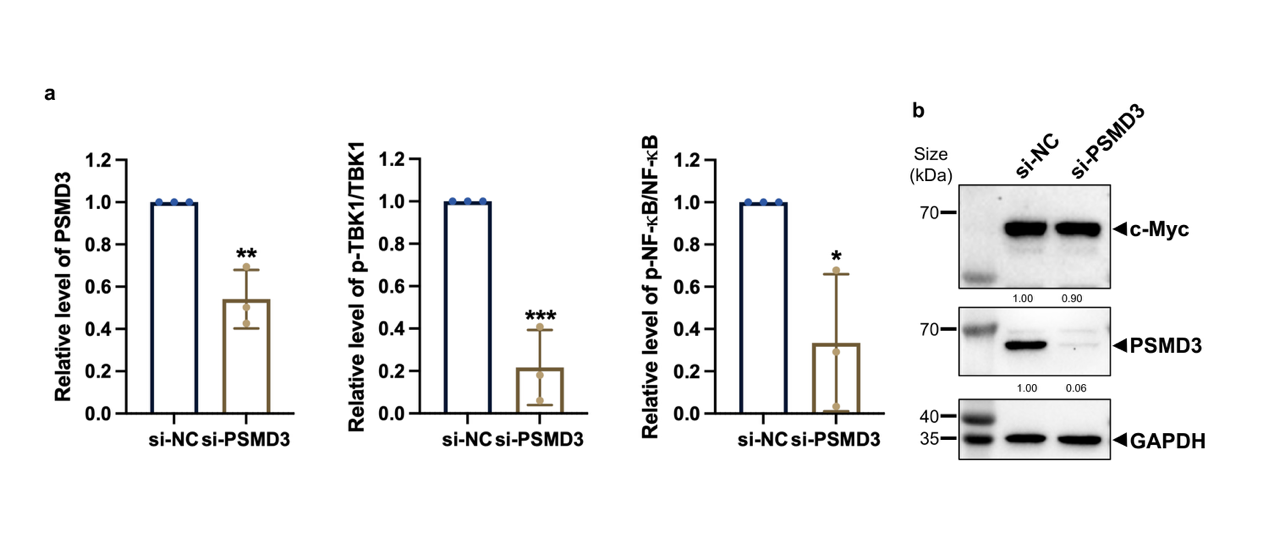


**Supplementary Fig. 10, related to Fig. 6: Knockdown of PSMD3 has no effect on c-Myc protein levels. a,** Related to Fig. 6a. Densitometric analysis of bands from three independent experiments was performed using ImageJ. Values were normalized against the corresponding internal control and presented relative to the si-NC group, and shown as mean ± SD. *p < 0.05, **p < 0.01, ***p < 0.001 by two tailed t-test. **b,** Knockdown of PSMD3 affects the protein level of endogenous c-Myc in MDA-MB-231 cells. WB analysis of the c-Myc protein levels affected by PSMD3 knockdown in MDA-MB-231 cells with indicated antibodies.


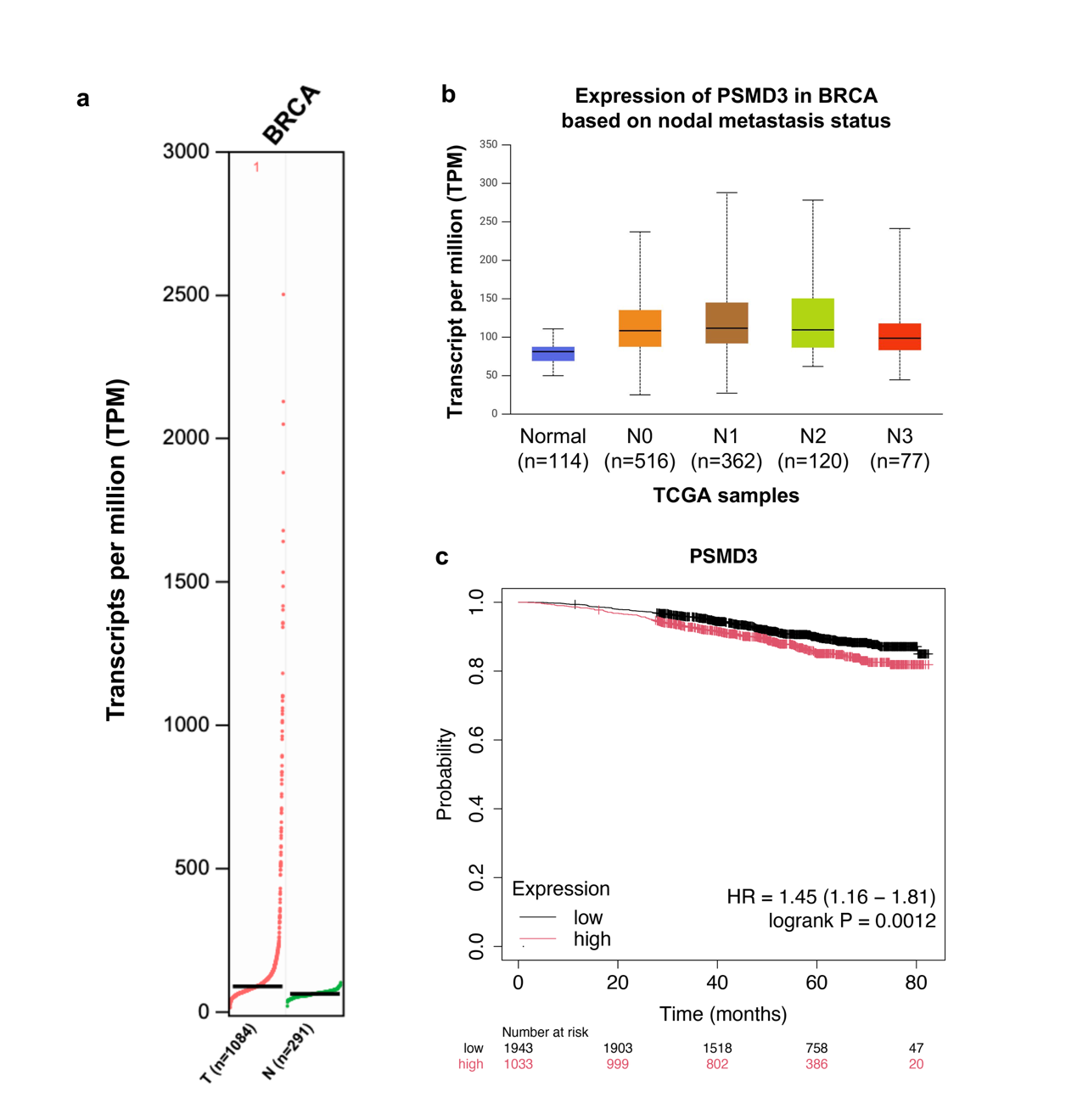


**Supplementary Fig. S11, related to Fig. 6: PSMD3 is highly expressed in aggressive and late-stage breast cancers. a,** Relative mRNA expression of PSMD3 in normal (n = 291) and breast cancer tissues (n = 1084) from the UALCAN BRCA cohort. **b,** Relative mRNA expression of PSMD3 in normal (n = 114) and breast cancer patients with different nodal metastasis statuses from the UALCAN Breast invasive carcinoma cohort. **c,** Kaplan-Meier analysis of PSMD3 expression and overall survival in the breast cancer cohort (n = 2976) from the Kaplan-Meier Plotter database. P value calculated by log-rank test.


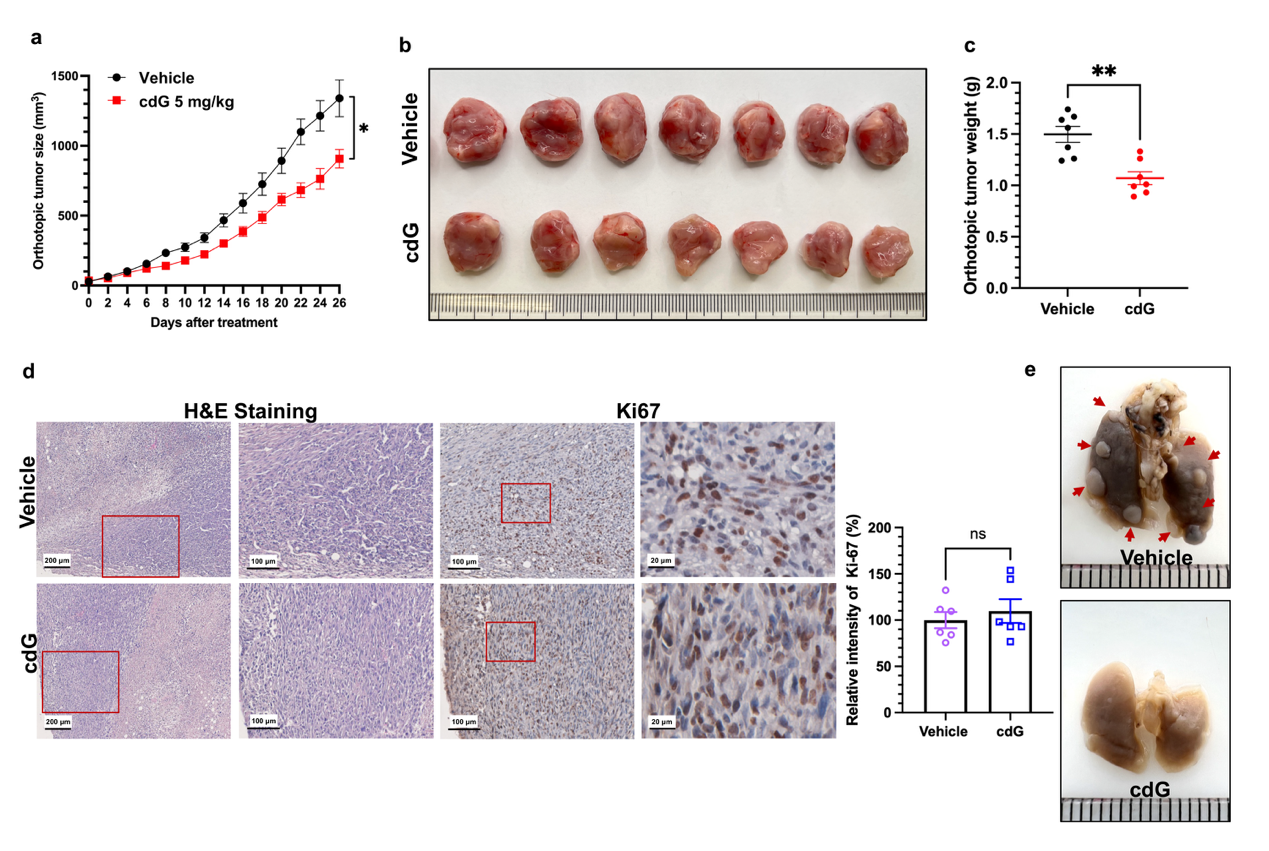


**Supplementary Fig. S12, related to Fig. 7: c-di-GMP inhibits lung metastasis in a mouse orthotopic mammary tumor model in vivo. a,** Tumor volume of orthotopic tumors measured at the indicated time points. Data represent mean ± SEM. *P < 0.05 by two-tailed Student’s *t*-test. **b,** Gross anatomy of final dissected orthotopic tumor masses (n = 7 per group). **c,** Tumor weights of the orthotopic tumor upon euthanasia at the endpoint. Each dot represents a tumor from an individual mouse. Data represent the mean ± SEM. **P < 0.01 by two-tailed Student’s *t*-test. **d,** H&E and IHC staining of orthotopic tumor sections from the indicated groups and analyzed using ImageJ/FIJI software. Data are presented as mean ± SEM. ns, not significant. Scale bar, 200 μm, 100 μm or 20 μm as indicated. **e,** Representative images of lung tumor nodules after formalin fixation.
