# Supplementary material for: Cyclic di-GMP suppresses cancer metastasis by targeting proteasome 26S subunit non-ATPase 3 independently of STING
Source: Signal Transduct Target Ther. 2026 Feb 4;11:44. doi: 10.1038/s41392-025-02553-9 (PMC12868630; doi:10.1038/s41392-025-02553-9)

Cyclic di-GMP suppresses cancer metastasis by targeting proteasome 26S subunit, non-ATPases 3 independently of STING

Jieqiong Wang^1,2^, Alexander Mrozek^1,2^, Kewen Hu^3,4^, Hanyu You^1,2^, Sarah E Traverse^1,2^, Hyemin Lee^1,2^, Shelya X. Zeng^1,2^, Xiufeng Pang^3^, Heewon Park^1,2^, Hua Lu^1,2,*^

Correspondence to: hlu2@tulane.edu

**This file contains all the raw western blot data related to each figure in the main figure set and supplementary figure set.**

**Raw Western blot data**


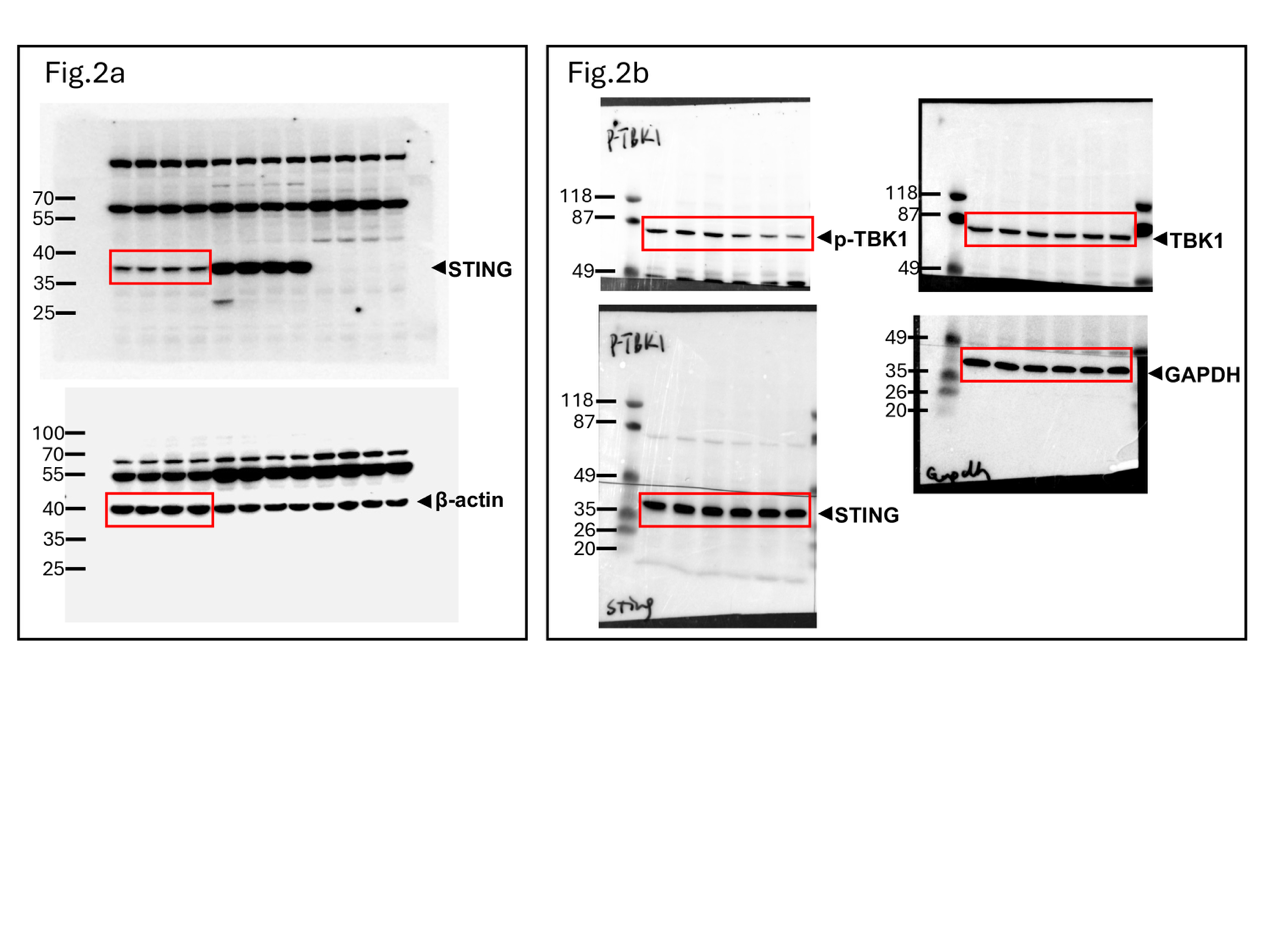


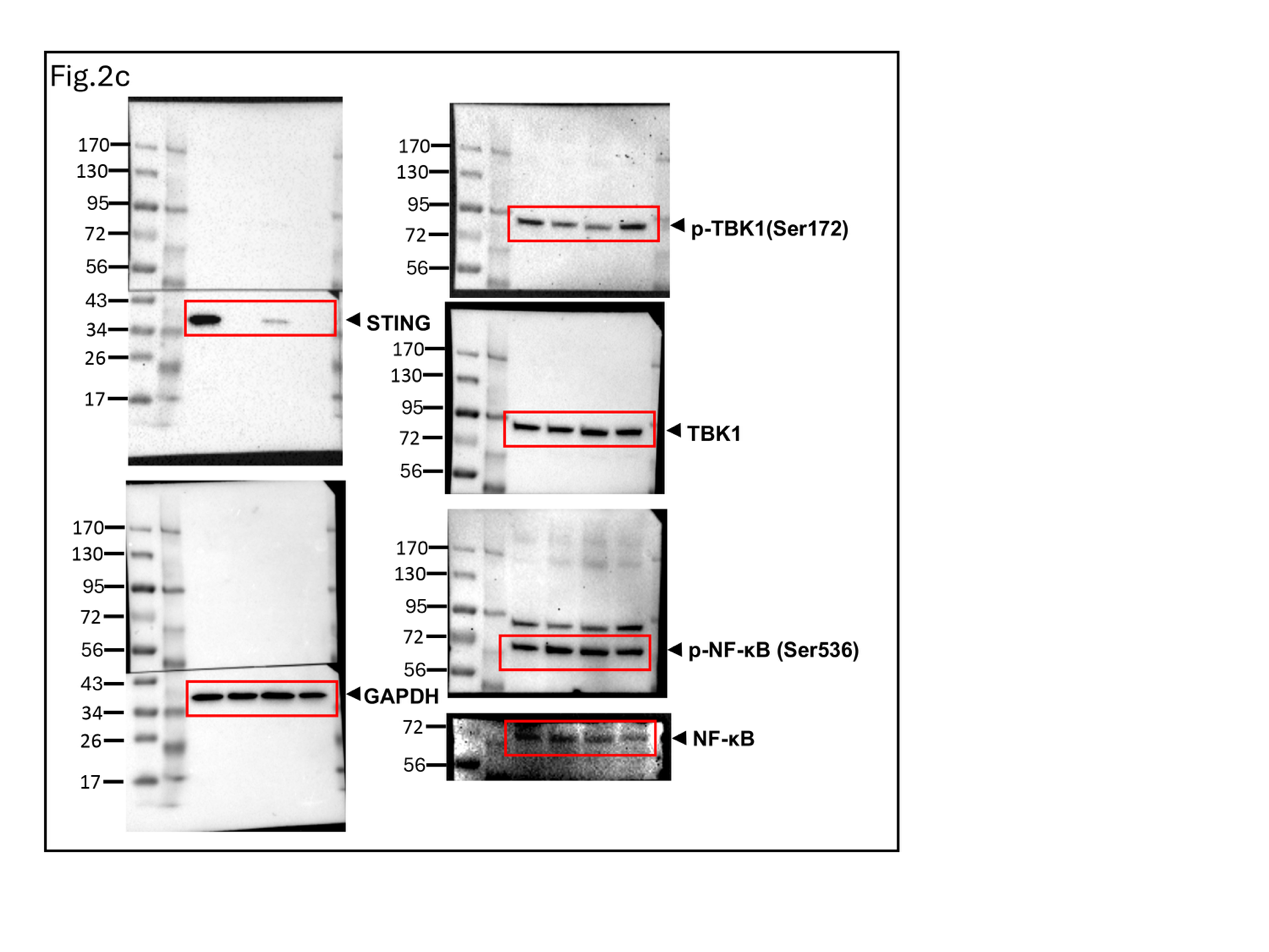


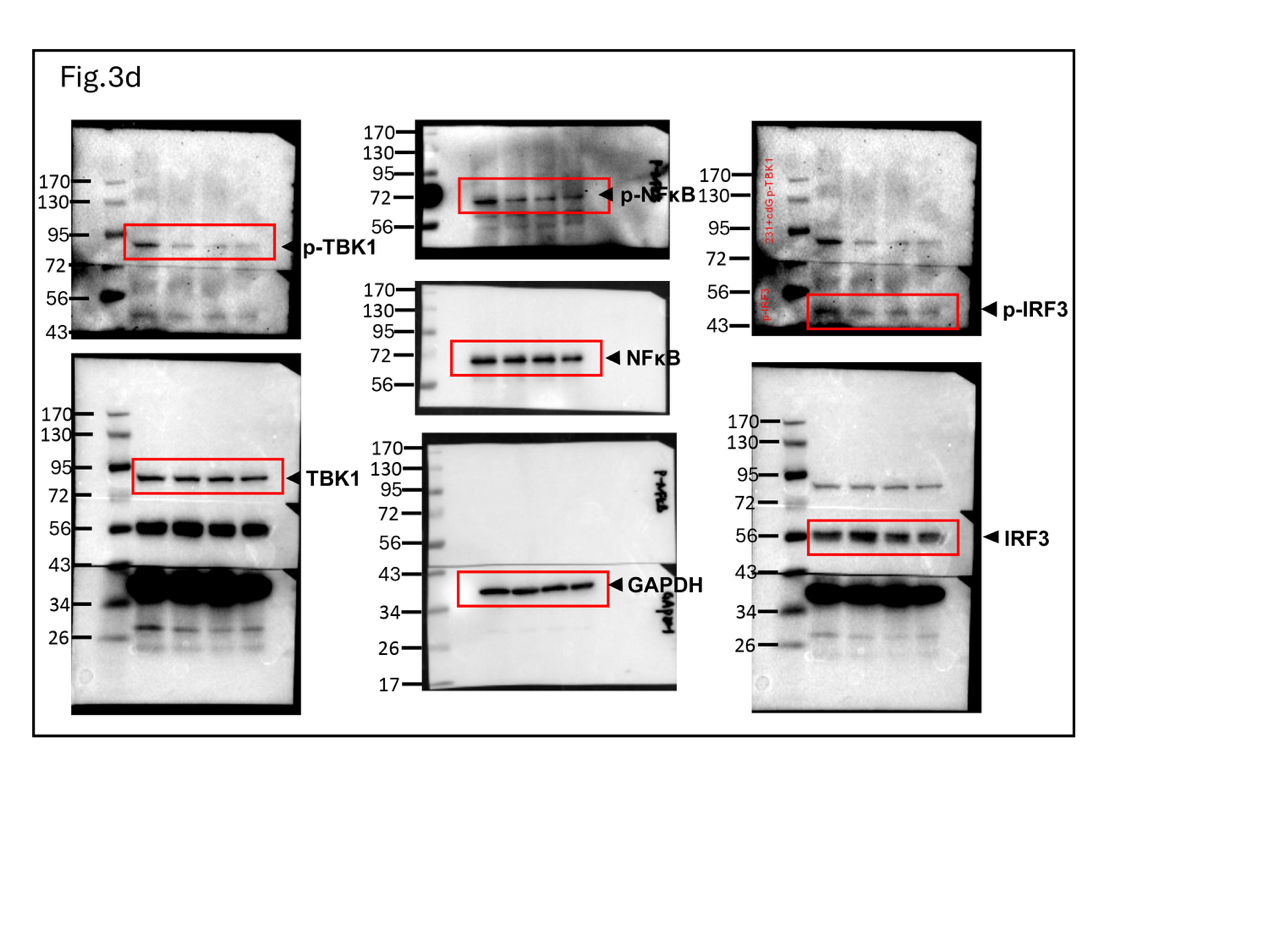


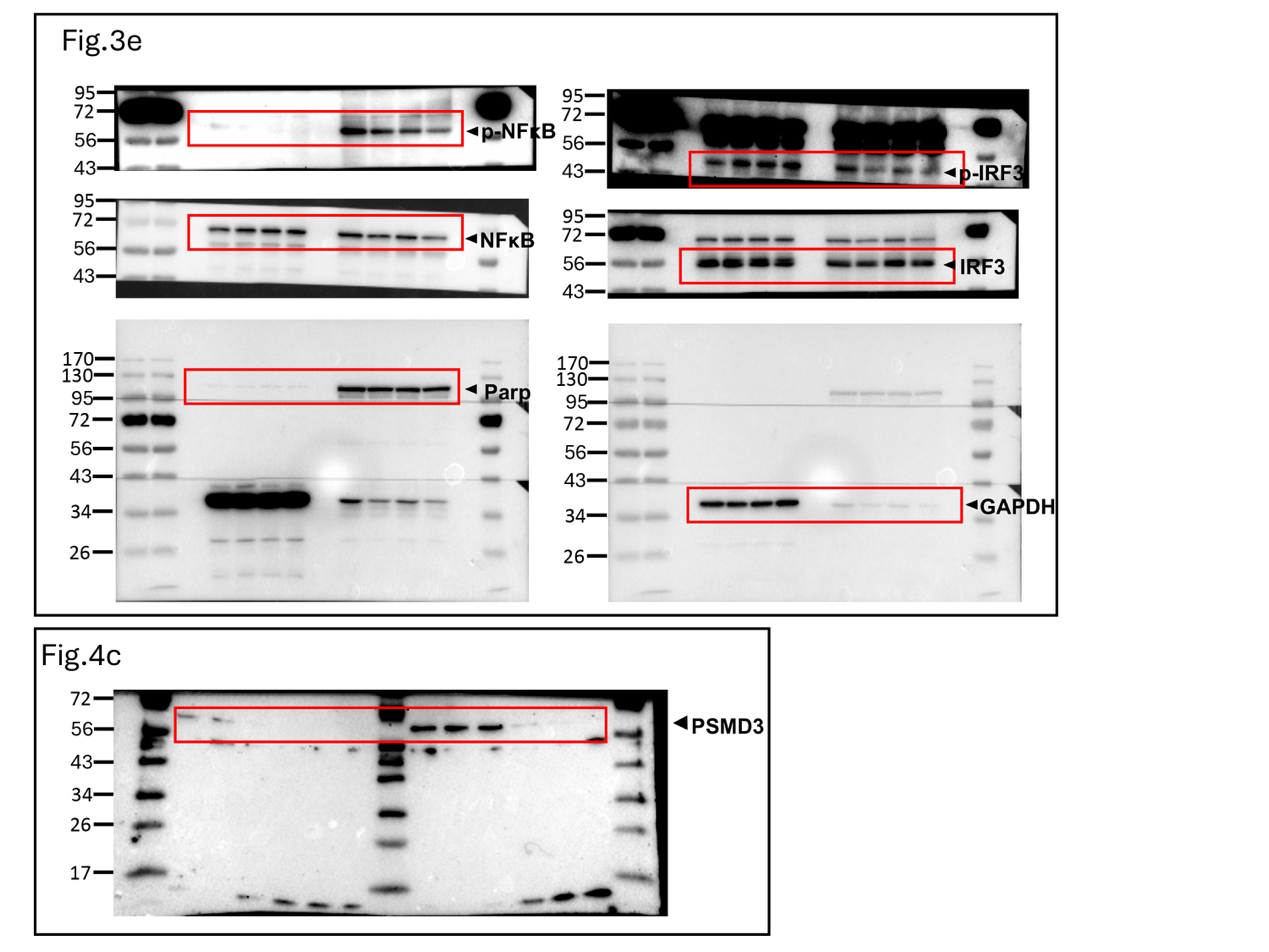


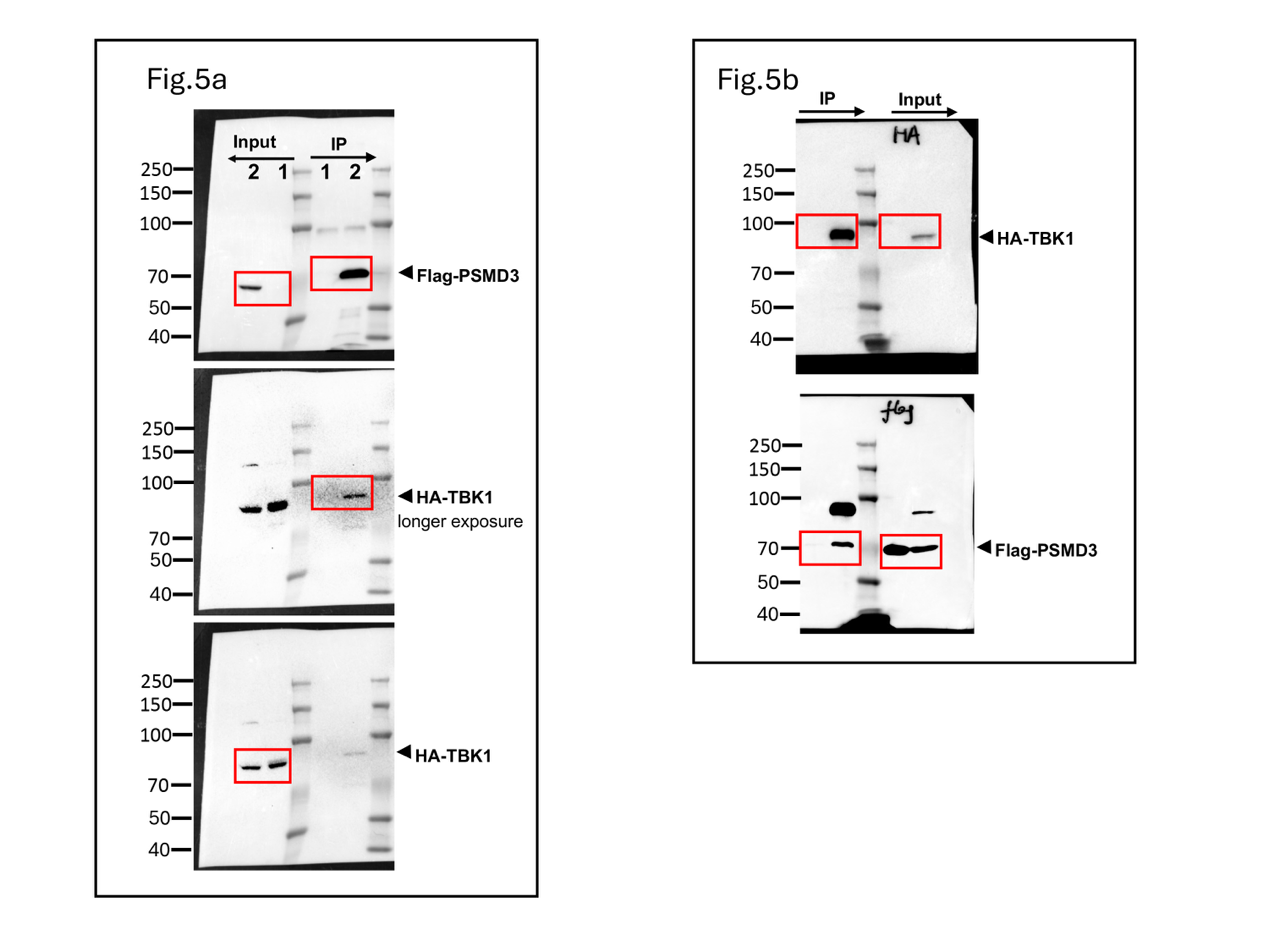


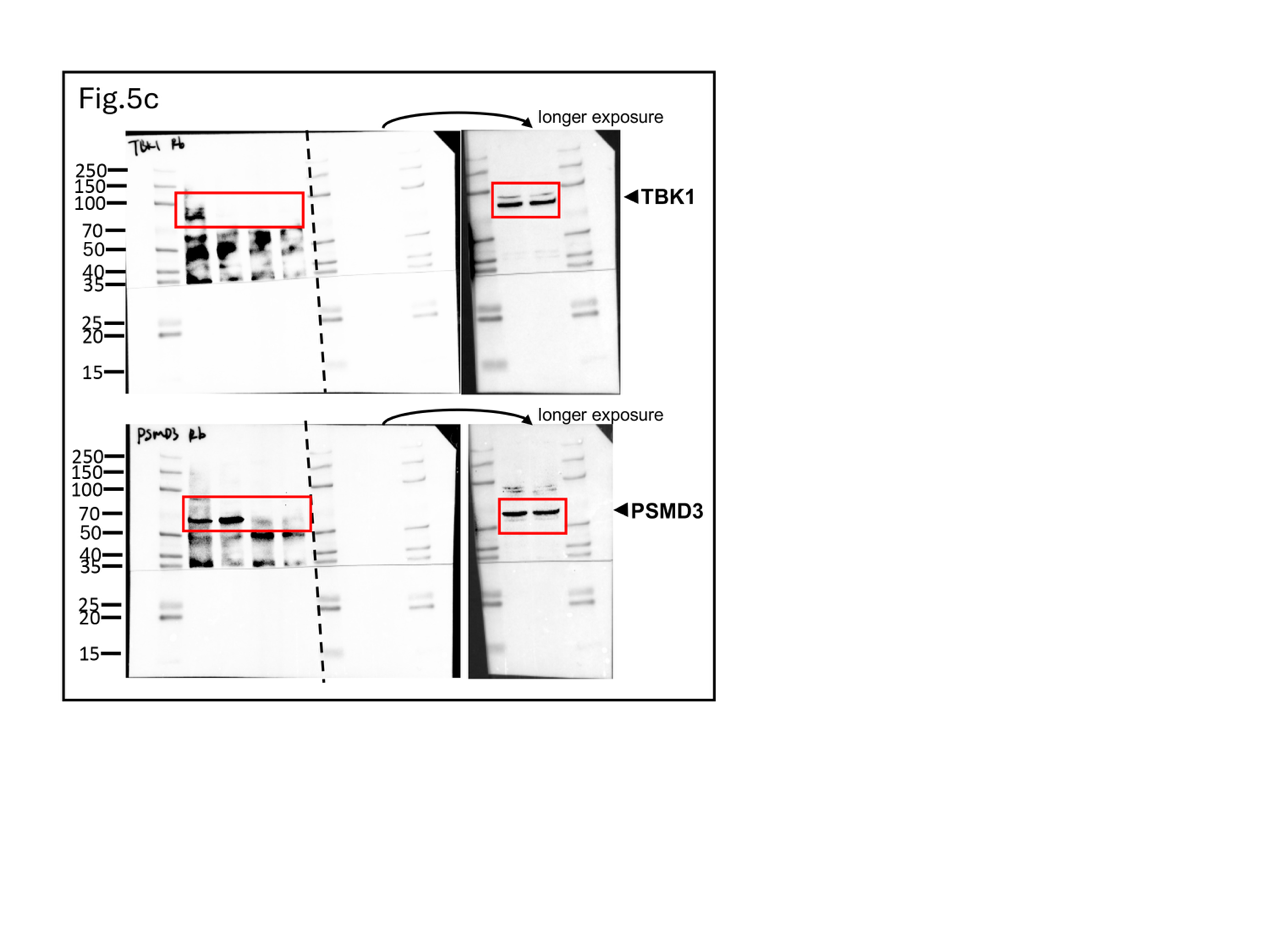


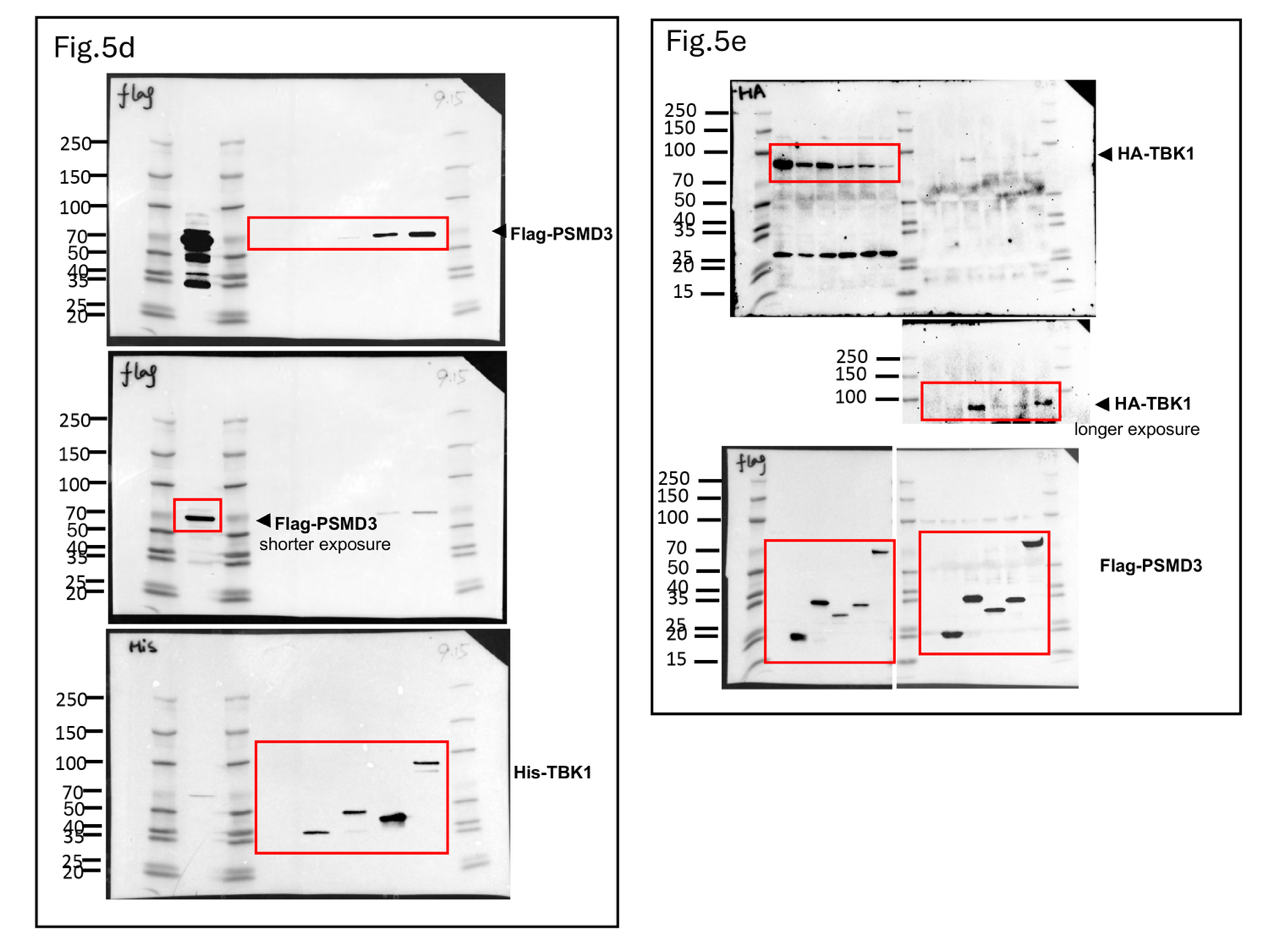


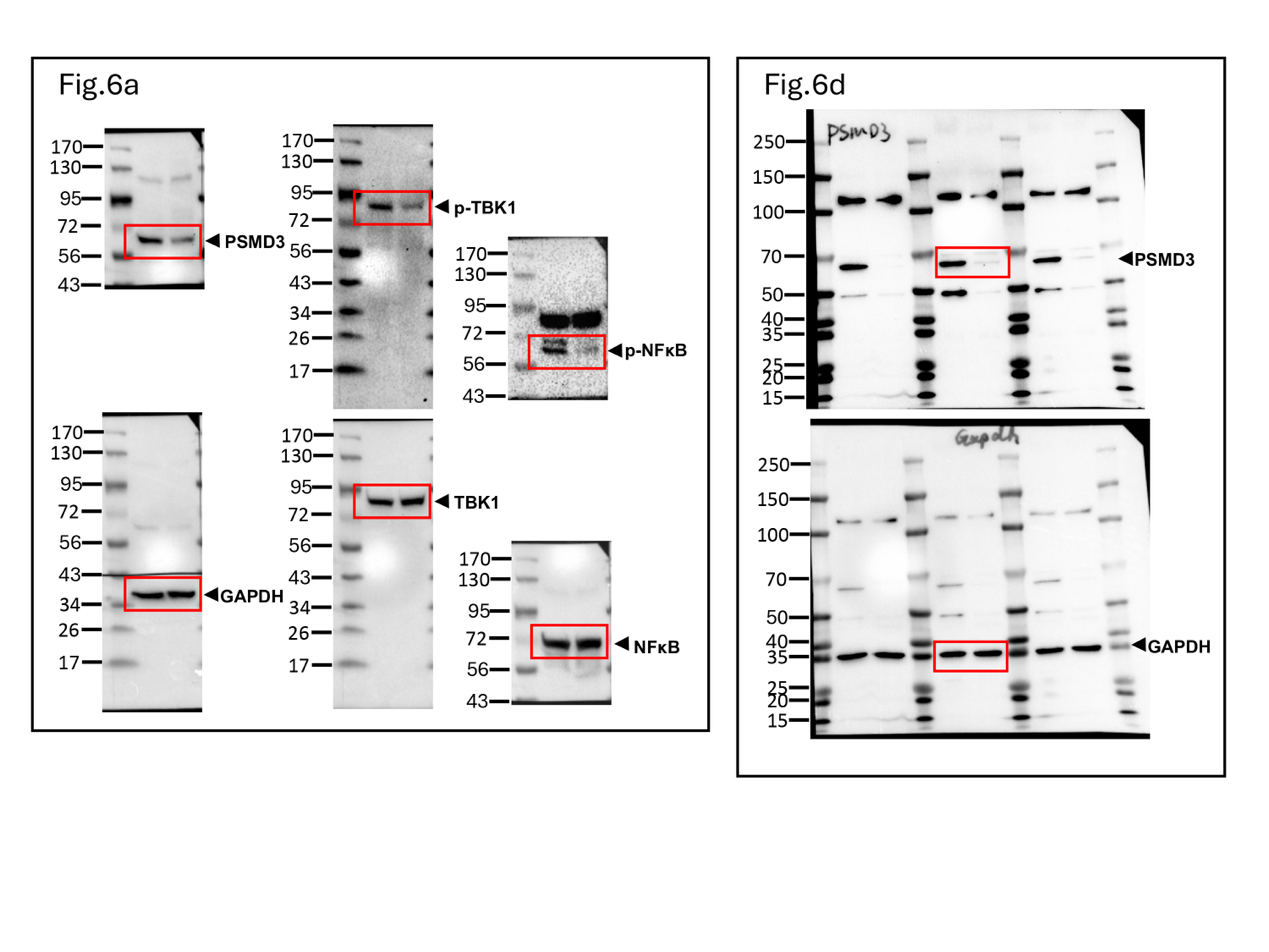


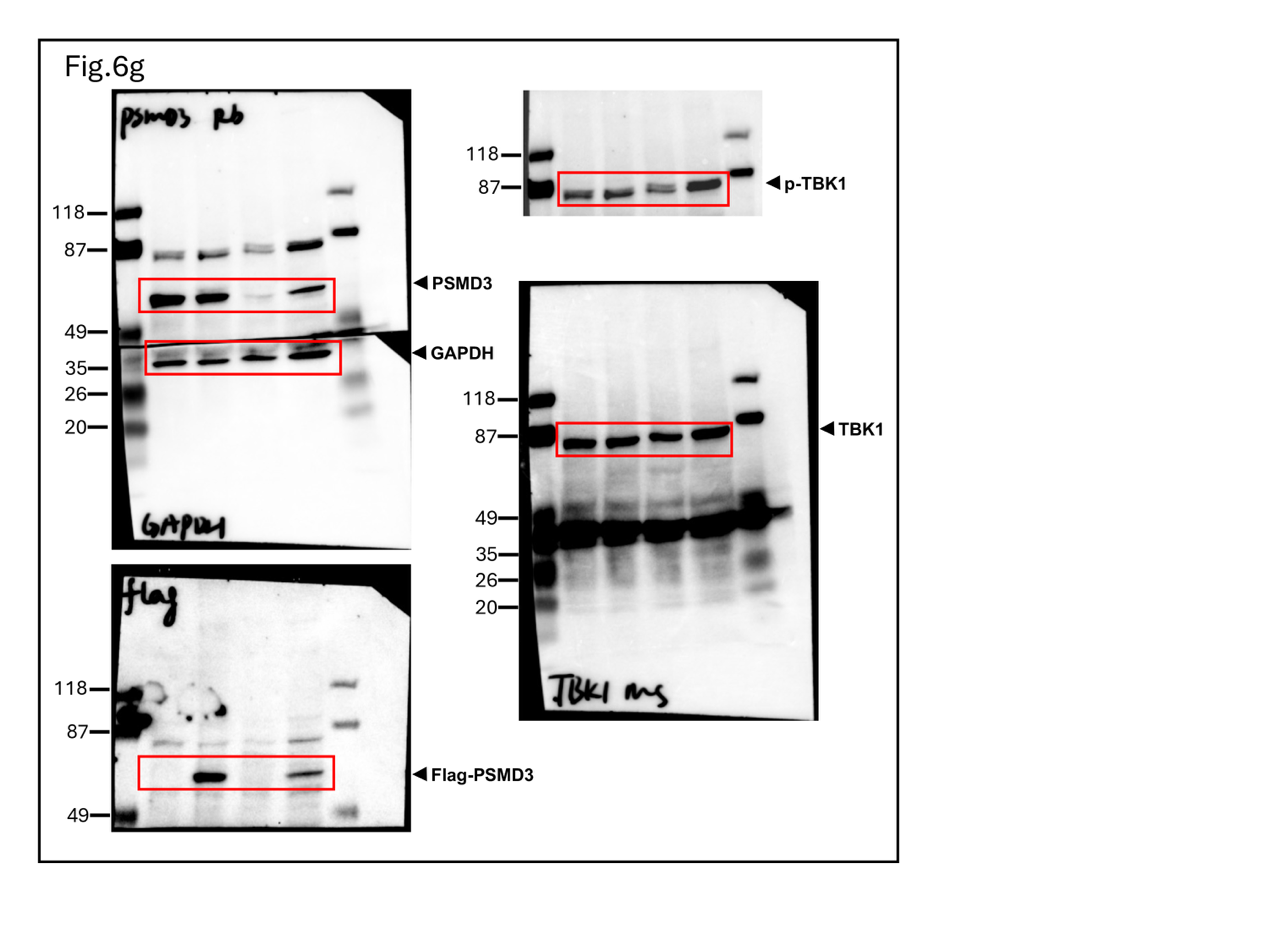


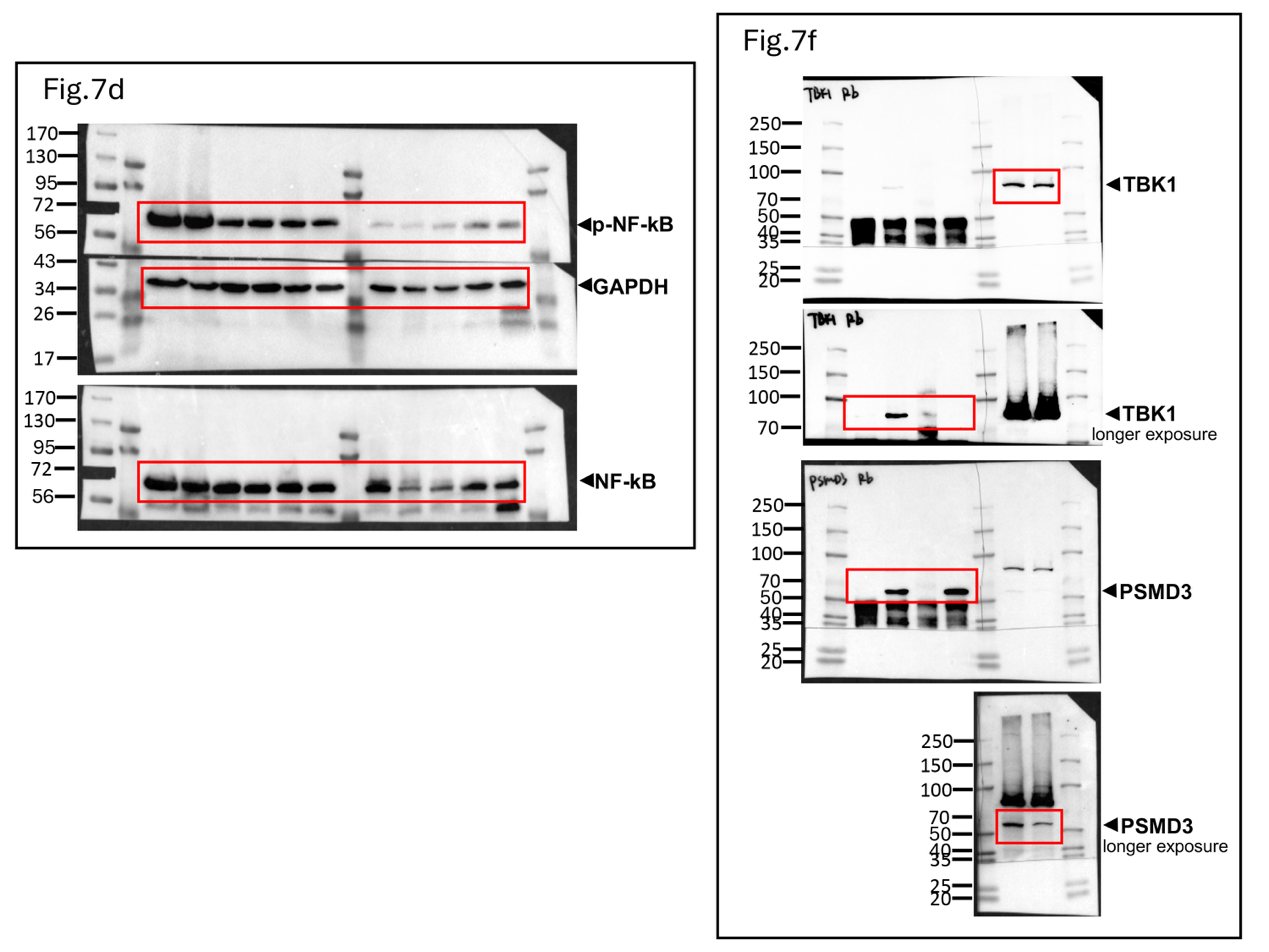


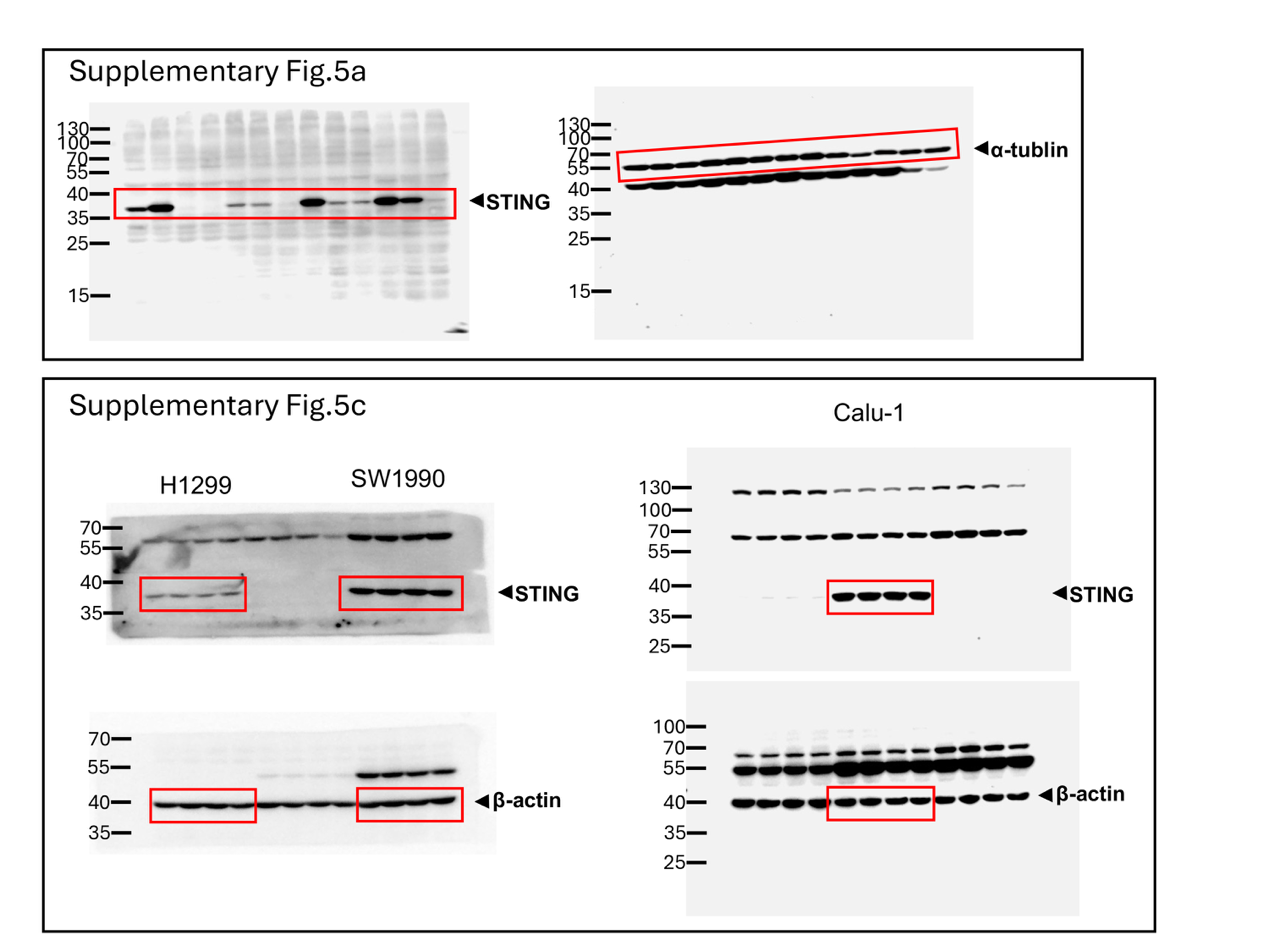


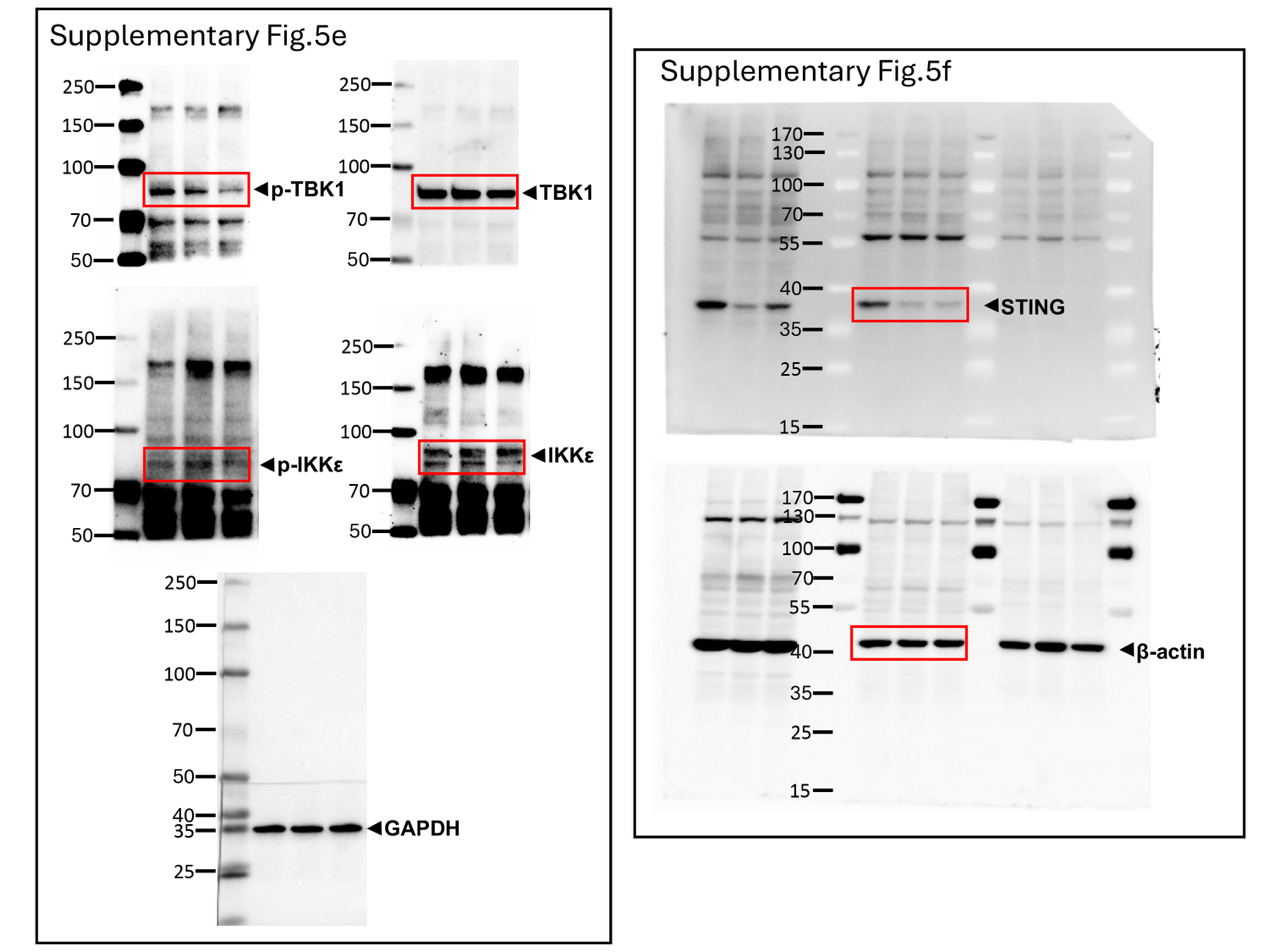


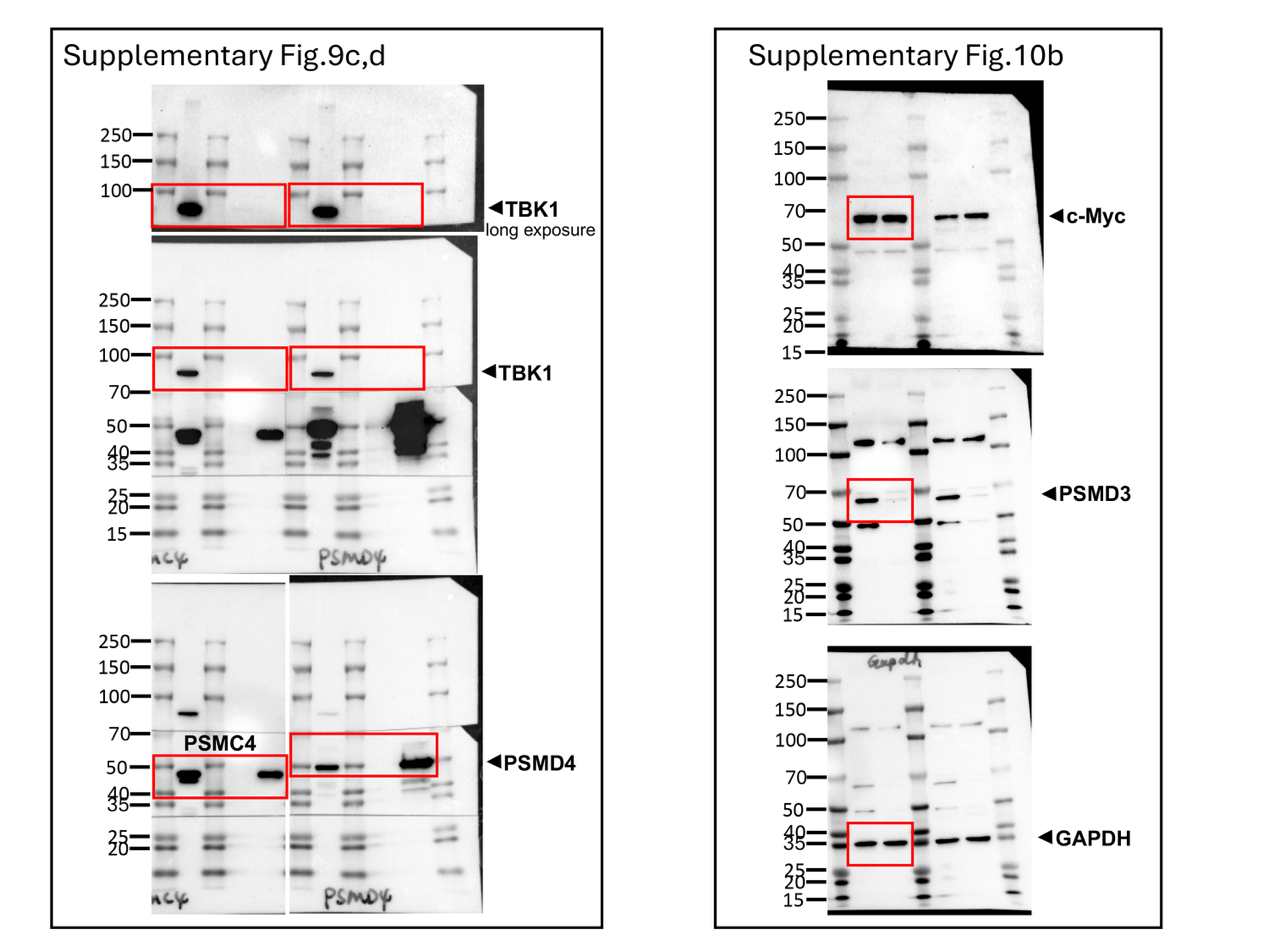

Supplement: Supplementary file 2 — Wang et al--Suppl raw WB blot data [file 41392_2025_2553_MOESM2_ESM.docx]
